# A Little Autonomy Support Goes a Long Way: Daily Autonomy‐Supportive Parenting, Child Well‐Being, Parental Need Fulfillment, and Change in Child, Family, and Parent Adjustment Across the Adaptation to the COVID‐19 Pandemic

**DOI:** 10.1111/cdev.13515

**Published:** 2021-01-19

**Authors:** Andreas B. Neubauer, Andrea Schmidt, Andrea C. Kramer, Florian Schmiedek

**Affiliations:** ^1^ DIPF Leibniz Institute for Research and Information in Education; ^2^ Center for Research on Individual Development and Adaptive Education of Children at Risk (IDeA); ^3^ Goethe‐University

## Abstract

This study examined the effects of daily parental autonomy support on changes in child behavior, family environment, and parental well‐being across 3 weeks during the COVID‐19 pandemic in Germany. Day‐to‐day associations among autonomy‐supportive parenting, parental need fulfillment, and child well‐being were also assessed. Parents (longitudinal *N* = 469; *M*_age_ *= *42.93, *SD*_age_ = 6.40) of school children (6–19 years) reported on adjustment measures at two measurement occasions and completed up to 21 daily online questionnaires in the weeks between these assessments. Results from dynamic structural equation models suggested reciprocal positive relations among autonomy‐supportive parenting and parental need fulfillment. Daily parental autonomy support, parental need fulfillment, and child well‐being partially predicted change in adjustment measures highlighting the central role of daily parenting for children’s adjustment during the pandemic.

The COVID‐19 pandemic in early 2020 has had a huge impact on virtually all individuals’ daily lives throughout the world. Shop closures, travel restrictions, and social distancing were but only some of the restrictions imposed by governments in various countries to slow down the spread of the Coronavirus and to avoid overburdening the national health care systems. The threat of the virus spread and its consequences on individual health, as well as the restrictions imposed to “flatten the curve” of new infections were likely not inconsequential for well‐being and psychological adjustment for most people, and therefore required psychological adaptation to this new situation. Families with school children were one group that has potentially been strongly affected by these measures (Prime, Wade, & Browne, 2020; United Nations Sustainable Development Group, 2020). School closings enforced by federal and local authorities in many countries left parents of school children with the task to take care of their children around the clock. For many parents, this implied that they would now have to balance work, household, child care, and potentially home schooling without help from their regular support systems (e.g., day‐care facilities or grandparents).

In the present work, we examined the adaptation of families with school children to the measures enforced to slow down the spread of the Coronavirus in Germany. With a longitudinal study, we examined change in parental well‐being, perceived family environment, and parent‐rated child behavior across 3 weeks during a time of school closings and other counter‐Corona measures (end of March until end of April 2020). Utilizing data from a 21‐day diary study embedded into this longitudinal study, we examined daily experiences—specifically, daily autonomy‐supportive parenting, parental need fulfillment, and child well‐being—as potential mechanisms driving change in these adjustment measures. Based on the classic conceptualizations of human development (Nesselroade, 1991), such short‐term processes might be the motor driving long‐term developmental change. Therefore, how families adapt to this situation has potentially important downstream consequences for both parents’ and children’s long‐term adjustment.

## Parenting and Children’s Psychological Adjustment

Parenting behavior has important consequences for children’s behavior and psychological adjustment. According to Self‐Determination Theory (SDT; Ryan & Deci, 2017), one critical dimension of parenting is autonomy support which entails, for example, taking the child’s perspective, avoiding controlling language (e.g., “must,” “should”) and offering meaningful choices when possible. Autonomy‐supportive parental behavior has been suggested to be associated with positive outcomes because it fosters intrinsic motivation and provides the opportunity for the child’s satisfaction of the needs for autonomy (the perception of self‐authorship of one’s actions), competence (being effective in one’s actions and attaining a sense of mastery), and relatedness (attaining a sense of belonging and relationships that are characterized by mutual caring; Ryan & Deci, 2017). In contrast, controlling parenting is characterized by coercive means and by using conditional regard, threats to punish, performance pressures, or guilt‐inducing criticisms as strategies to control their child’s behavior (Mageau et al., 2015). Controlling parenting behavior has been suggested to thwart children’s basic needs and thereby to lead to problematic behavior (e.g., internalizing or externalizing behavior) and negative downstream consequences such as loss in academic motivation and psychopathology (see Soenens & Vansteenkiste, 2010). Experiencing the environment as actively thwarting one’s needs has been referred to as need frustration. Notably, prior theoretical (Vansteenkiste & Ryan, 2013) and psychometric work (e.g., Neubauer & Voss, 2018) suggests that need satisfaction and need frustration are not mere psychometric opposites but should be considered as related but distinct constructs with separable antecedents and consequences. For example, while need satisfaction has been primarily linked to psychological adjustment such as vitality and life satisfaction, need frustration is more directly linked to negative outcomes such as depression (Chen et al., 2015).

In the present work, we will focus on autonomy‐supportive parenting as a type of parental behavior that has been associated with positive outcomes such as autonomous motivation (Grolnick, Raftery‐Helmer, Flamm, Marbell, & Cardemil, 2015), prosocial behavior (Rueth, Otterpohl, & Wild, 2017), and well‐being (e.g., van der Kaap‐Deeder, Vansteenkiste, Soenens, & Mabbe, 2017). We chose to target autonomy‐supportive parenting rather than controlling parenting in this work in order to identify potentially beneficial behaviors that could support the adaptation of families during the COVID‐19 pandemic and hence inform potential interventions to facilitate adaptation. Summarizing prior research on autonomy‐supportive parenting, a meta‐analysis of 36 correlational studies (Vasquez, Patall, Fong, Corrigan, & Pine, 2016) including samples from preschool to college age reported an average correlation of *r* = .36 between parental autonomy support and children’s psychological health (i.e., well‐being, self‐esteem). In addition, longitudinal studies support the prediction that autonomy‐supportive behavior is associated with changes in children’s well‐being and behaviors (Duineveld, Parker, Ryan, Ciarrochi, & Salmela‐Aro, 2017; Joussemet, Koestner, Lekes, & Landry, 2005). There is also abundant research that has examined the positive effects of autonomy‐supportive parenting on adaptive outcomes in adolescents (e.g., Lekes, Gingras, Philippe, Koestner, & Fang, 2010; Won & Yu, 2018) and emerging adults (e.g., Inguglia et al., 2016). This suggests that, in line with SDT’s universality claim, autonomy‐supportive parenting is important for psychological growth and well‐being from childhood to adolescence and beyond (Ryan & Deci, 2017).

Autonomy‐supportive parenting is hypothesized to positively impact children’s adjustment by providing children with the necessary conditions for the fulfillment of the psychological needs for autonomy, competence, and relatedness. These positive short‐term effects may have potentially beneficial downstream consequences on a larger timescale, for example, by impacting general aspects of well‐being via a bottom‐up pathway of more positive momentary experiences (similar to a broaden‐and‐build perspective; Fredrickson, 2001). Positive effects on academic achievement and other behavioral outcomes (Joussemet et al., 2005) may, however, also be mediated via a motivational pathway: According to SDT, autonomy‐supportive parenting also fuels the internalization of motivation (Ryan & Deci, 2017), hence yielding more autonomously motivated behavior, which in turn is associated with the fulfillment of basic psychological needs (Hope, Holding, Verner‐Filion, Sheldon, & Koestner, 2019). In this way, autonomy‐supportive parenting may set off a positive developmental cascade culminating in psychological growth, integrity, and well‐being (Ryan & Deci, 2017). In addition, autonomy‐supportive parenting could strengthen the parent–child relationship and thus foster children’s attachment to their parents. Secure attachment has been linked not only to child well‐being, but also to positive child behavior (e.g., prosocial behavior; Shaver, Mikulincer, Gross, Stern, & Cassidy, 2016).

Hence, there are several pathways by which autonomy‐supportive parenting might positively impact both short‐term and long‐term developmental outcomes. In the present study, we therefore examined if parental autonomy support during the COVID‐19 pandemic was associated with both better daily child well‐being, and with increases in positive child behavior (prosocial behavior) and decreases in negative child behavior (emotional problems, hyperactivity) across 3 weeks.

## Effects of Autonomy‐Supportive Parenting on the Family Environment

There is some research suggesting that autonomy‐supportive parenting has beneficial effects not only for the child toward whom the behavior is targeted, but also for other family members. A study by van der Kaap‐Deeder et al. (2015) followed pairs of siblings in a daily diary study for 5 consecutive days and collected ratings on daily perceived autonomy support from their mother, and perceived autonomy support from their sibling. Findings suggested that the amount of maternal autonomy support perceived by one sibling was associated with higher amount of sibling autonomy support reported by the other sibling. Hence, children who perceived their mothers as more autonomy supportive were perceived as more autonomy supportive by their siblings. This suggests that autonomy‐supportive parenting might not only influence the experience and behavior of the child toward whom parenting behavior is targeted, but might also affect the social network around this child (e.g., the family). We targeted this prediction by examining the effects of autonomy‐supportive parenting on longitudinal change in perceptions of the family environment.

According to Moos (1990), family environment is a multidimensional construct encompassing three broad aspects: family relationships, system maintenance, and personal growth. In the present work, we targeted the first two of these aspects. Specifically, positive emotional climate (i.e., a construct encompassing open expression of thoughts and feelings, cohesion, and individual freedom; Roth, 2002), was expected to be the dimension of family environment most susceptible to the influence of autonomy‐supportive parenting. Providing children with individual choices might aid in building an environment that facilitates an open and positive family climate. Hence, autonomy‐supportive parenting provides the basis for positive emotional climate, with support provision and the perception of autonomy support provided by other family members constituting a key factor for the development of a positive emotional climate. In contrast, the aspect of system maintenance according to Moos (1990), which encompasses the dimensions of control (a family environment with clear communication of obligations and values) and organization (characterized by, e.g., thorough planning of common family activities), is likely more resistant to change in a short term.

## Autonomy‐Supportive Parenting and Parental Well‐Being

Autonomy‐supportive parenting might also yield beneficial effects on parental well‐being. In a different interpersonal context (friendships), findings from a study by Deci, La Guardia, Moller, Scheiner, and Ryan (2006) suggest that providing autonomy support to a friend is associated with higher well‐being for the support provider. In the realms of parenting, recent studies have shown that autonomy‐supportive parenting is positively associated with parental need fulfillment (Dieleman et al., 2019; Mabbe, Soenens, Vansteenkiste, van der Kaap‐Deeder, & Mouratidis, 2018; van der Kaap‐Deeder et al., 2015, 2019). In these studies, it has been suggested that need fulfillment fuels autonomy‐supportive parenting via reductions in parental stress and increases in parental vitality: Autonomy‐supportive parenting is resource intensive and requires energy on part of the parents. Need fulfillment provides energy and thereby supplies parents with the necessary resources to provide their children with autonomy support. So far it is, however, unclear, if need fulfillment fuels autonomy support, if parental need fulfillment is a consequence of autonomy‐supportive parenting, or if the two constructs are reciprocally related (an issue on which we elaborate in the next section). If autonomy‐supportive parenting has (unidirectional or reciprocal) effects on subsequent parental need fulfillment, this could further lead to positive changes in parental well‐being. In addition, reductions in problematic child behavior and improvements in perceived family environment as a consequence of autonomy‐supportive parenting could also feed back into improvements in parental well‐being.

Taken together, there is some evidence suggesting positive effects of parental autonomy support not only on indicators of child adjustment, but also on well‐being of the provider of autonomy support and on the family environment more generally. In the present work, we examined if parental autonomy support would be associated with more positive development of family climate and parental well‐being across 3 weeks.

## Day‐to‐day Dynamics of Parental Autonomy Support and Parental Need Fulfillment

Recent findings suggest that autonomy‐supportive parenting behavior is not a fixed trait across time, but changes on a shorter timescale (e.g., from day to day). Children perceive their parents’ level of autonomy‐supportive behavior differently on a day‐to‐day basis, and these short‐term fluctuations in autonomy‐supportive behavior have been linked to children’s need fulfillment and well‐being on a daily level: On days when children perceived their mother’s behavior as more autonomy‐supportive than usual, they also reported higher levels of need satisfaction and positive affect (PA), and slightly lower levels of need frustration (van der Kaap‐Deeder et al., 2017). Similarly, parents’ reports of their autonomy‐supportive behavior also vary from day to day and these fluctuations have been linked to parental need fulfillment (Dieleman et al., 2019; Mabbe et al., 2018; van der Kaap‐Deeder et al., 2019).

However, the temporal association between parental autonomy support and parental need fulfillment is somewhat unclear. Previous diary studies have reported same‐day associations (Dieleman et al., 2019; Mabbe et al., 2018; van der Kaap‐Deeder et al., 2019). That is, days on which parents reported higher need fulfillment were also days on which they reported more autonomy‐supportive behavior. As noted by Mabbe et al. (2018), the association between parental need fulfillment and autonomy support is, however, likely reciprocal, with both constructs reinforcing each other across time. While across‐day effects among some of the relevant constructs have been examined in prior work (e.g., the across‐day associations among autonomy support and parental well‐being; van der Kaap‐Deeder et al., 2019), no prior study has examined the across‐day effects of need fulfillment on autonomy support on the next day. To illuminate the potentially reciprocal relation between daily autonomy‐supportive parenting and daily parental need fulfillment, we examined their interplay across 3 weeks using dynamic structural equation models (DSEMs; Asparouhov, Hamaker, & Muthén, 2017). These models combine elements of multilevel models, time‐series analysis, and structural equation models and they have been proposed and implemented as an approach to examine the within‐person lead‐lag associations among multiple variables (Hamaker, Asparouhov, Brose, Schmiedek, & Muthén, 2018).

## The Present Study

We examined the potentially beneficial effects of autonomy‐supportive parenting on changes in perceived child behavior, family environment, and parental well‐being across 3 weeks in a sample of parents of school children in Germany. Over these 3 weeks, strict measures to slow down the spread of the Coronavirus had been enforced by the German government including travel restrictions, measures enforcing social distancing, and school closings. We examined if, during these challenging times, autonomy‐supportive parental behavior would facilitate adaptation, as indicated by less child problem behavior, a more positive family environment, lower parental stress levels, and higher parental vitality. Furthermore, we illuminated the hypothesized, but so far untested, reciprocal relation between autonomy‐supportive parenting and parental need fulfillment. We also tested whether autonomy‐supportive parenting would be associated with better child well‐being. Using DSEMs, we examined across‐day effects among autonomy‐supportive parenting, parental need fulfillment, and child well‐being. Finally, we examined if parental need fulfillment, children’s daily well‐being, and parental autonomy support would be uniquely related to longitudinal changes in adaptation.

Taken together, we tested the following hypotheses in confirmatory analyses: (a) Daily autonomy‐supportive parenting will be associated with higher parental need fulfillment and better child well‐being on the same day. (b) Parental autonomy support will be positively and reciprocally related to parental need satisfaction across days. Furthermore, parental autonomy support will predict child well‐being on the next day. (c) Change in family environment, child behavior, and parental well‐being will be related to parental autonomy support, with more autonomy‐supportive parenting being associated with increases in positive emotional climate, children’s prosocial behavior, and parental vitality, and with decreases in children’s emotional problems and hyperactivity as well as parental stress. (d) Above and beyond autonomy‐supportive parenting, daily child well‐being will be related to changes in child behavior (prosocial behavior, emotional problems, hyperactivity), and daily parental need satisfaction (frustration) will predict changes in parental vitality (stress) across 3 weeks.

## Method

The PACO study (Psychological Adjustment to the COVID‐19 pandemic) was conducted to examine the adjustment of parents of school children to the restrictions imposed in response to the spread of the Coronavirus. The study was approved by the local ethics committee.

### Participants and Procedure

Participants for this online study were recruited via posting study information on social media platforms, contacts to schools and parent–teacher associations, as well as a press release issued by the authors’ institution. The baseline questionnaire (an online questionnaire implemented via soscisurvey.de) was published on March 27, 2020, and deactivated 1 week later (April 3). During these 8 days, 1,206 participants started the questionnaire and provided informed consent. We excluded participants who terminated the study before all sociodemographic variables were queried (approximately 20% of the online questionnaire). This resulted in a final sample for the baseline questionnaire of 970 participants (838 women; *M*_age_ = 42.93, *SD*_age_ = 6.40, range = 25–82). The inclusion criteria for participation were (a) age 18 years or older and (b) currently cohabiting with at least one child who attends school in the current school year. Criterion 1 was assessed as part of the informed consent; Criterion 2 was assessed with an item asking for the number of currently cohabiting schoolchildren. Most participants were married (*N* = 647; 66.7%) or in a permanent relationship and living in one household with their partner (*N* = 116; 12.0%). One hundred and fourteen (11.8%) were divorced, 53 (5.5%) single, 28 (2.9%) in a permanent relationship living in separate household, and 11 (1.1%) widowed. Net monthly household income was above 4,000€ for 446 participants (46.0%); 237 participants (24.4%) reported a monthly household income below 3,000€, and 208 (21.4%) between 3,000€ and 4,000€. Seventy‐nine participants (8.1%) did not report their household income.

Most (*N* = 810; 83.5%) of these 970 participants finished the baseline questionnaire. At the end of the questionnaire, participants could sign up for the second part of this study: a daily diary study over the next 21 days followed by a post assessment the day after the last day of the diary period. A total of 562 participants signed up for this second part; 469 participants provided data at the post assessment. Due to a technical error, one participant from the second study part could not be matched to his or her baseline data. We compared participants who continued with the second study part to those who did not on all demographic variables and relevant baseline measures reported in this manuscript. None of the 21 tests (depending on the variable either independent *t*‐test or chi‐square‐test) were statistically meaningful, *p* > .091 for all (see Table S1 for all comparisons).

In the daily diary part, participants received an e‐mail with the link to the daily questionnaire each day at 7 p.m., which was deactivated at 5 a.m. the following morning. All 562 participants completed at least one of the daily questionnaires, and in total 7,747 daily questionnaires were (at least partially) completed across the 21 days. This corresponds to a compliance rate of 65.6% (7,747/(562 × 21)). Seventy‐four participants did not complete any questionnaires after the first week was over. Not considering these 13.2% dropouts yielded a compliance of 73.8% (7,558/(488 × 21)) for those participants who continued the daily part of the study for more than 1 week. Participants did not receive direct remuneration for participation in this study, but they could enter a lottery. Participants in the first study part (baseline assessment) could win one of the 40 retail vouchers of 50€ each. In the second study part, 100 retail vouchers (50€ each) and three iPads were raffled among all participants. For each questionnaire in the daily diary that was filled in, participants received one ticket to this lottery (two tickets for the post questionnaire).

### Measurements

Only the measures relevant for the present work are described here (see Table S2 for an overview); a more detailed account of the study and all study variables (including additional references to other studies that have used these measures) is presented in the study protocol, which can be found together with all data and analysis code necessary to reproduce the results reported here on the OSF repository (https://osf.io/aj6fk/). Descriptive statistics of the main study variables can be found Table S3.

All child‐related variables reported in this study were assessed for the youngest school‐aged child in the participants’ household. That is, if only one child lived in the participants’ household, participants were asked to answer the items with respect to this child. If multiple children lived in their household, they were instructed to rate the items with respect to the youngest school‐aged child in their household. Target children were on average 9.81 years old (*SD* = 2.85, range = 6–19); 500 target children were male, 460 female, 3 were diverse/nonbinary. Most children (*n* = 610; 62.9%) attended elementary school, 240 children (24.7%) the academic tier of secondary school (German *Gymnasium*), 20 children (2.1%) attended a basic or vocational secondary school, 53 (5.5%) a comprehensive school, and 44 children (4.5%) a different school type.

#### Baseline and Post Measures

Four instruments that were assessed at the baseline assessment and the post assessment are relevant for the context of the present study.

##### Child behavior

We administered three subscales of the Strengths and Difficulties Questionnaire (SDQ; Goodman, 1997; Klasen et al., 2000) to assess parents’ perceptions of the youngest school‐aged child in their household. The three subscales were *emotional problems*, *hyperactivity/inattention*, and *prosocial behavior*. The subscales *conduct problems* and *peer problems* were not administered given that social distancing measures rendered these subscales less relevant in the present context. Participants were asked to rate for each of the 15 statements to what extent they were true for the child with respect to the last week (0 = not true; 1 = somewhat true; 2 = completely true). For each scale, five items were averaged and multiplied by 5, yielding a potential range of each scale from 0 to 10. Reliability was estimated as internal consistency (McDonald’s ω) and was adequate in the present study: emotional problems (ω_Baseline_ = .75; ω_Post_ = .79); hyperactivity/inattention (ω_Baseline_ = .83; ω_Post_ = .84); prosocial behavior (ω_Baseline_ = .74; ω_Post_ = .76). Prior work has demonstrated that parent reports on the SDQ converge with reports by other sources such as self‐reports obtained from adolescents (Becker, Hagenberg, Roessner, Woerner, & Rothenberger, 2004) and correlations with related measures such as the Child Behavior Checklist (Muris, Meesters, & van den Berg, 2003) supporting the validity of the SDQ.

##### Family environment

A German adaptation (Roth, 2002) of the Family Environment Scale (Moos & Moos, 1981) was administered in this sample. Specifically, we used 19 items that have previously been used in adolescents to measure the dimensions positive family environment, organization, and control. Participants were presented with 19 statements (e.g., “Each member of our family has the same rights when decisions are made.”; “We have to improvise a lot because nothing is really planned.”) and asked to rate to what extent these statements applied to their family in the last week on a scale from 1 (*not at all true*) to 5 (*completely true*). As these items have not been used in prior research with parents yet, we conducted exploratory and confirmatory factor analyses to investigate their factor structure. Based on these results (see Supporting Information S4 and Tables S5 and S6 for details), 17 items of this scale were retained. These items built four factors: organization, control, family cohesion, and expressiveness. The latter two dimensions correspond to the positive family environment component of the family environment scale.

##### Stress

Parents’ stress was assessed with the 10‐item version of the Perceived Stress Scale (Cohen, Kamarck, & Mermelstein, 1983). Participants read 10 statements (e.g., “Last week, I was upset because of something that happened unexpectedly.”) and asked to indicate to what extent they agree with it on a scale ranging from 1 (*completely disagree*) to 7 (*completely agree*). All items referred to experiences in the last week. Responses were averaged into one perceived stress score (ω_Baseline_ = .88; ω_Post_ = .89).

##### Vitality

The Subjective Vitality Scale (Goldbeck, Hautzinger, & Wolkenstein, 2019; Ryan & Frederick, 1997) was administered. Participants rated for seven items (e.g., “I feel alive and vital.”) to what extent they agree with these statements when considering their current situation. Internal consistencies were satisfactory (ω_Baseline_ = .93; ω_Post_ = .93). The German adaptation of this scale has recently been validated and exhibited the theoretically expected associations with, for example, depression, anxiety, and vigor, as well as sensitivity to change after a brief walking intervention (Goldbeck et al., 2019).

#### Daily Measures

Only the measures relevant for the present study are reported here (see associated repository for a complete description of all variables).

##### Autonomy‐supportive parenting

Two items were administered to measure autonomy‐supportive parenting toward the target child (the youngest school‐aged child in the household) today: “As far as possible, I let my child decide today what he or she wanted to do.”; “As far as possible, my child was able to do what he or she liked today.” These two items were chosen to capture one central aspect of autonomy support: choice within certain limits. The items were adapted from similar assessment approaches in prior cross‐sectional (Mageau et al., 2015) and daily diary studies (Mabbe et al., 2018). The two items were meaningfully correlated on the within‐person level, *r* = .67, and the between‐person level, *r* = .86, and they were averaged into one autonomy support indicator per day and participant.

##### Child well‐being

Participants were asked to rate their child’s well‐being today. Eight items were administered to assess negative affect (NA; four items: afraid, angry, sad, and worried) and PA (four items: happy, cheerful, balanced, and relaxed). Participants were instructed to rate the extent to which their child (again referring to the youngest school‐aged child in the household) experienced these affective states today on a scale from 1 (*not at all*) to 7 (*very*). Reliability for the two subscales was estimated separately for the within‐person level and the between‐person level via a multilevel extension of McDonald’s ω (Geldhof, Preacher, & Zyphur, 2014). Internal consistencies were estimated as ω_Between_ = .87/ω_Within_ = .66 (NA), and ω_Between_ = .91/ω_Within_ = .84 (PA).

##### Parental need fulfillment

The daily diary version (Neubauer & Voss, 2018) of the revised Balanced Measure of Psychological Needs Scale (Neubauer & Voss, 2016; Sheldon & Hilpert, 2012) was used to measure participants’ daily need fulfillment. This scale can be used to measure daily need fulfillment as a six‐dimensional construct, with fulfillment of the three needs for autonomy, competence, and relatedness split into their satisfaction and their frustration components. Prior work using this scale has reported the associations of need satisfaction and need frustration with indicators of well‐being and ill‐being (Koehler & Neubauer, 2020; Neubauer & Voss, 2018). Internal consistencies were satisfactory on both levels of analyses for all six subscales, autonomy satisfaction (ω_Between_ = .89/ω_Within_ = .73), competence satisfaction (.94/.76), relatedness satisfaction (.97/.78), autonomy frustration (.90/.72), competence frustration (.91/.62), and relatedness frustration (.91/.71). Since we did not have expectations for specific needs and were interested in need satisfaction and frustration, respectively, across need dimensions, we averaged the three satisfaction scales into one overall need satisfaction score per day and person, and the three frustration scales into one overall need frustration score per day and person.

#### Covariates

In all models we included time‐varying covariates that were assessed on each day of the daily diary: the time parents reported spending with their child today (in hours), whether their child worked on materials or tasks for school today (coded 0 for not, and 1 for yes), and parents’ daily Corona‐related worries (the mean of two items: “Today, I spent a lot of time thinking about the corona pandemic.”; “Today, I worried about the corona pandemic and its effects on myself and my family.”). In addition, we included day of the week (0 = during the week; 1 = weekend or holiday) of the daily assessment as additional covariate. Furthermore, we controlled for several person‐level covariates in the analyses: target child’s age and gender, parent gender, the number of children living in the household, expected change in household income, participants’ employment situation, as well as parents’ baseline depressive symptoms (assessed with the Center for Epidemiologic Studies Depression Scale; Hautzinger, 1988) and loneliness (assessed with a short form of the UCLA loneliness scale; Hays & DiMatteo, 1987). Expected change in household income was computed as the difference between current monthly net household income and participants’ expected net household income in the upcoming months (both were assessed in 500€ increments). Participants’ employment situation was entered as a factor with three levels (unemployed, working from home, and working from outside home), transformed into two dummy variables with working from home as the reference category. Finally, for the analyses predicting change across 3 weeks, we controlled for how many days after the end of the Easter vacation participants completed the post assessment. Since the timing of Easter vacation varies by state, we determined this variable via participants’ zip code (assessed in the baseline assessment). The difference between the day of post assessment and the last day of the Easter vacation in days was computed for each participant.

### Data Analysis

#### General modeling choices

Structural equation models were estimated in Mplus version 8.3 (Muthén & Muthén, 1998–2020) using the robust maximum likelihood estimator. Model fit was evaluated based on conventional cutoff criteria for absolute fit indexes (comparative fit index [CFI] > .90; root mean square error of approximation [RMSEA] < .08; standardized root mean square residual [SRMR] < .08). For all analyses, a conventional alpha level of .05 (two‐tailed) was assumed. For the DSEM analysis, which operates in a Bayesian framework, we considered effects as statistically meaningful when their 95% credible interval did not contain zero.

#### Multilevel Models and DSEMs

We examined the within‐person association of autonomy‐supportive parenting with parental need fulfillment and child well‐being both within days and across days. For the within‐day associations, we used multilevel models with daily observations (Level 1) nested within participants (Level 2). In the first model, autonomy‐supportive parenting on day *d* was predicted by need satisfaction and need frustration on the same day. These two predictors were centered on the person mean to estimate the unconfounded within‐person effects (Wang & Maxwell, 2015). The resulting within‐person effects indicate if days on which participants reported, for instance, higher need satisfaction than they do on average were also days on which they reported higher‐than‐usual autonomy‐supportive behavior. Mean levels of need satisfaction and need frustration (centered on their grand means) were entered to examine the between‐person effects (targeting the question if, e.g., participants who reported more need satisfaction on average also reported more autonomy‐supportive behavior across the 21 days). The time‐varying covariates time spent with children today, Corona‐related worries, and homeschooling were entered as Level‐1 predictors (centered on the respective person mean) and Level‐2 predictors (the person means centered on the grand means). Weekend was entered as a dummy predictor comparing weekend days to week days. On the between‐person level, we controlled for target child’s age and gender, parent gender, the number of children living in the household, expected change in household income, participants’ employment situation, parents’ baseline depressive symptoms, and loneliness. Random effects for need satisfaction and need frustration were estimated and random effects were allowed to correlate. In the second (third) model, daily child PA (NA) was predicted by daily autonomy‐supportive parenting (a time‐varying predictor centered on the person mean) and the individual’s average autonomy‐supportive parenting score as a person‐level predictor (centered on the grand mean). Models were estimated using the R package nlme (Pinheiro, Bates, DebRoy, Sarkar, & R Core Team, 2019). In these models, data from all participants who completed at least one daily assessment and who had complete data on covariates were included (*N* = 497).

To examine across‐day effects, we set up DSEMs in Mplus version 8.3. In these models, all observations are first decomposed into a between‐person component and a within‐person component. On the within‐person level, the five variables autonomy support, need satisfaction, need frustration, child PA, and child NA were entered into a cross‐lagged model, indicating that all five variables were predicted by these five variables on the previous day (all auto‐ and cross‐regression effects). Random effects were estimated for the 20 cross‐regression effects. Covariates in this model were day of the week (during the week vs. weekend), time spent with children today, Corona‐related worries, and homeschooling. Because we were exclusively interested in the within‐person day‐to‐day associations in this model, we fitted a fully saturated model on the between‐person model (i.e., allowing the five variables and four covariates to freely correlate on Level 2) to reduce model complexity. For the analyses, we kept the Mplus defaults for (uninformative) priors, and used two chains with 3,000 iterations, burn‐in period of 50%, and thinning of 100. Results are therefore based on the posterior distribution of 3,000 draws.

#### Latent Change Score Model

To determine the amount of change in the constructs from baseline to post assessments, a latent change score model was estimated, which reparameterizes the structural model into a model of true change (McArdle, 2009). With this approach, mean level change and interindividual differences in intraindividual change can be assessed. This model was then used in a multilevel structural equation model to predict interindividual differences in change in family environment, child behavior, and parental well‐being from daily experiences. In Model 1, autonomy support (modeled as latent variable with two indicators on both the between‐ and the within‐person level) was added as a predictor of the change variables on the between‐person level. Furthermore, we controlled for baseline levels of the respective change variables (a change regression model sensu McArdle, 2009). Correlations among baseline levels and the focal predictor (autonomy support) were estimated. In Model 2, daily parental need satisfaction and need frustration, and daily child PA and NA were entered as additional predictors.

## Results

### Daily Dynamics Among Autonomy‐Supportive Parenting, Parental Need Fulfillment, and Child Well‐Being

We first examined if there was evidence for average change across the 21 study days in the five variables (autonomy support, need satisfaction, need frustration, child PA, and child NA) in separate multilevel models. There was no effect of study day on autonomy‐supportive behavior, *b* = .000, *p* = .988, parental need satisfaction, *b* = −.000, *p* = .821, parental need frustration, *b* = −.002, *p* = .231, or child PA, *b* = −.001, *p* = .668, but a slight decrease in child NA, *b* = −.007, *p* < .001. Results from multilevel models with need satisfaction and need frustration predicting autonomy‐supportive behavior on the same day revealed that on days when parents reported higher need satisfaction, *b* = .137, *p* < .001, and lower need frustration, *b* = −.191, *p* < .001, they also reported engaging in more autonomy‐supportive behavior (for full results see Table S7). On the between‐person level, participants who reported more average need satisfaction, *b* = .246, *p* < .001, and less average need frustration, *b* = −.178, *p* = .001, reported more autonomy‐supportive behavior on average across the 21 days. Children’s daily PA (NA) was reported as higher (lower) on days when parents reported more autonomy‐supportive behavior, *b* = .277, *p* < .001 (*b* = −.176, *p* < .001). Furthermore, parents who reported more autonomy‐supportive behavior on average, also reported higher (lower) average levels of PA (NA) in their children, *b* = .314, *p* < .001 (*b* = −.185, *p* < .001). Together, findings suggest positive associations among (parent‐reported) autonomy‐supportive behavior, parental need fulfillment, and child well‐being on both the within‐person level and the between‐person level, supporting our first research hypothesis.

We next targeted across‐day associations with DSEM using data from all participants who completed at least two daily assessments (*N* = 535). The two chains of the DSEM analyses converged successfully, as indicated by a maximum probability of scale reduction = 1.003. Visual inspection of the trace plots revealed successful mixing and no irregularities. Complete results of the DSEM analyses are reported in Table S8. All five autoregressive effects were statistically meaningful, indicating that today’s autonomy support (need satisfaction, need frustration, PA, NA) predicted tomorrow’s autonomy support (need satisfaction, need frustration, PA, NA). In line with our second research hypothesis, findings suggested mutually reinforcing effects of autonomy‐supportive behavior and need satisfaction: days with higher autonomy‐supportive behavior were followed by days with more positive change in need satisfaction, *b* = .030, [.008, .051], β = .043 [.012, .072], and days with higher need satisfaction were followed by days with more positive change in autonomy‐supportive behavior, *b* = .054 [.008, .100], β = .036 [.007, .065]. That is, when parents were more autonomy‐supportive than usual on a given day, they reported an increase in need satisfaction on the next day, and vice versa. There were no reliable across‐day associations of autonomy‐supportive behavior with need frustration, child PA, or child NA. Hence, contrary to our expectations, autonomy‐supportive parenting had no effect on child well‐being on the next day.

### Change in Adjustment Measures

Before examining change across the 3 weeks, we conducted a confirmatory factor analysis for all nine scales (family cohesion, expressiveness, organization, control, emotional problems, hyperactivity/inattention, prosocial behavior, vitality, and perceived stress) using the data from the whole sample of the baseline questionnaire (see Supporting Information S4 and Table S6 for additional details on the individual measurement models). Model fit was acceptable, χ^2^(1,087) = 2,555.689, RMSEA = .037, and SRMR = .050, CFI = .908. Table 1 depicts the correlations among the latent factors in this model (see Table S9, for standardized factor loadings).

**Table 1:** Factor Variances and Correlations

	Variance	2	3	4	5	6	7	8	9
1. Cohesion	0.263*	.561*	.325*	.059	−.090*	−.250*	.479*	.316*	−.307*
2. Expressiveness	0.340*		.282*	−.006	−.122*	−.101*	.186*	.190*	−.182*
3. Organization	0.766*			.480*	−.206*	−.236*	.249*	.283*	−.258*
4. Control	0.414*				−.034	−.030	.133*	.191*	−.094*
5. Emotional problems	0.047*					.407*	−.219*	−.332*	.438*
6. Hyperactivity	0.237*						−.531*	−.397*	.398*
7. Prosocial behavior	0.146*							.320*	−.347*
8. Vitality	1.648*								−.670*
9. Stress	1.371*								

Note

Estimated variances and correlations of the latent factors in the baseline sample. *N* = 962.

* *p* < .05.

For all models, we assumed strict measurement invariance across time. For detailed results on the tests for measurement invariance, please see Supporting Information S10 and Table S11. Results of the latent difference models are reported in Table 2 (for correlations among the latent variables in this model see Table S12). Results are based on data by all participants who took part at both the baseline assessment and the post assessment (*N* = 468). Findings showed that only two of these scales exhibited significant average changes across the 3 weeks: Participants reported increased prosocial behavior of their children across the 3 weeks and a decrease in the family environment subscale control. Notably, there were statistically meaningful interindividual differences in change in all variables, as evidenced by the variances of the latent change variables (see Table 2). In the next step, we examined daily autonomy‐supportive behavior as a potential predictor of these changes. Note that, given the model complexity, we split up the model into three separate models and examined change in the four family environment scales in one model, change in the three SDQ subscales in a second model, and change in parental well‐being (vitality and stress) in a third model.

**Table 2:** Latent Difference Score Model: Means and Variances of Latent Variables

	*M*	Variance
Cohesion	3.802 (.031)*	0.285 (.030)*
Change cohesion	0.030 (.025)	0.068 (.024)*
Expressiveness	4.534 (.029)*	0.286 (.045)*
Change expressiveness	−0.033 (.026)	0.117 (.030)*
Organization	3.477 (.039)*	0.549 (.044)*
Change organization	−0.029 (.024)	0.107 (.023)*
Control	3.127 (.032)*	0.353 (.032)*
Change control	−0.050 (.023)*	0.077 (.017)*
Emotional problems	0.416 (.020)*	0.142 (.016)*
Change emotional problems	−0.012 (.018)	0.088 (.014)*
Hyperactivity	0.821 (.025)*	0.233 (.016)*
Change hyperactivity	0.005 (.017)	0.067 (.011)*
Prosocial behavior	1.476 (.019)*	0.127 (.011)*
Change prosocial behavior	0.033 (.015)*	0.039 (.009)*
Vitality	4.204 (.061)*	1.617 (.089)*
Change vitality	0.014 (.054)	1.215 (.108)*
Stress	3.810 (.059)*	1.431 (.086)*
Change stress	−0.078 (.050)	0.887 (.093)*

Note

Table displays means and variances of the latent variables in the latent difference score model (*SE*s in parentheses). See Table S12 for correlations among these variables. *N* = 468.

* *p* < .05.

Results are reported in Tables 3 and 4 (Model 1). Regarding family environment, daily autonomy‐supportive parenting was associated with an increase in parents’ perceived cohesion, β = .412, *p* < .001. Autonomy‐supportive behavior was unrelated to changes in the other three family environment scales (*p* > .217 for all). Contrary to expectations, autonomy‐supportive behavior did not predict changes in children’s emotional problems, β = −.017, *p* = .792, hyperactivity, β = −.159, *p* = .079, or prosocial behavior, β = .043, *p* = .601. When considering change in parental well‐being, daily provided autonomy support was associated with increasing vitality, β = .141, *p* = .009, but unrelated to parents’ reported stress, β = −.062, *p* = .144. Hence, our third research hypothesis was only partially supported by the data.

**Table 3:** Latent Change Regression Models: Predicting Change in Family Environment From Daily Autonomy Support, Daily Parental Need Fulfillment, and Daily Child Well‐Being

Predictor	Cohesion		Expressiveness		Organization		Control	
	Model 1	Model 2	Model 1	Model 2	Model 1	Model 2	Model 1	Model 2
Baseline level	−.326 (.114)*	−.387 (.135)*	−.223 (.142)	−.234 (.148)	−.104 (.087)	−.100 (.089)	−.052 (.105)	−.045 (.111)
Autonomy support	.412 (.121)*	.306 (.137)*	.027 (.077)	−.002 (.089)	.119 (.101)	.125 (.118)	.119 (.096)	.065 (.122)
Parental need satisfaction	—	.207 (.122)	—	.201 (.093)*	—	.008 (.123)	—	−.045 (.135)
Parental need frustration	—	−.105 (.136)	—	−.173 (.111)	—	.067 (.119)	—	.027 (.130)
Child PA	—	.212 (.149)	—	−.077 (.128)	—	−.026 (.140)	—	.093 (.150)
Child NA	—	.048 (.147)	—	.139 (.124)	—	−.139 (.129)	—	−.133 (.142)
Covariates								
Corona‐related worries	−.166 (.128)	−.132 (.125)	−.133 (.082)	−.129 (.084)	−.111 (.089)	−.090 (.090)	.125 (.097)	.128 (.100)
Homeschooling	−.130 (.125)	−.058 (.117)	−.104 (.092)	−.112 (.095)	−.145 (.099)	−.117 (.101)	−.137 (.102)	−.113 (.104)
Time spent with child	.274 (.098)*	.247 (.094)*	.261 (.066)*	.248 (.067)*	.186 (.086)*	.201 (.085)*	−.063 (.095)	−.069 (.095)
Parent gender	−.110 (.084)	−.150 (.089)	−.073 (.073)	−.094 (.074)	.118 (.068)	.116 (.067)	−.075 (.070)	−.069 (.069)
Target child’s gender	.002 (.094)	.005 (.090)	−.109 (.067)	−.102 (.067)	−.053 (.075)	−.068 (.078)	.009 (.082)	.008 (.083)
Target child’s age	.107 (.120)	.161 (.123)	−.036 (.089)	−.063 (.084)	.127 (.086)	.128 (.088)	−.142 (.097)	−.130 (.100)
Number of children in household	−.215 (.096)*	−.186 (.094)*	−.129 (.077)	−.102 (.077)	−.076 (.070)	−.085 (.073)	.059 (.083)	.046 (.084)
Parent employment: work away from home	−.038 (.097)	−.109 (.095)	.003 (.077)	.002 (.078)	−.033 (.075)	−.056 (.075)	.133 (.086)	.115 (.090)
Parent employment: no employment	.039 (.098)	.004 (.089)	.066 (.074)	.070 (.075)	.005 (.083)	−.006 (.082)	−.014 (.080)	−.035 (.082)
Expected change in income	.140 (.139)	.118 (.134)	.235 (.075)*	.203 (.074)*	.089 (.077)	.087 (.075)	−.029 (.100)	−.013 (.098)
Days end of holidays to post assessment	−.062 (.074)	−.043 (.074)	.026 (.048)	.033 (.049)	.139 (.068)*	.138 (.065)*	−.009 (.086)	−.008 (.091)
Loneliness	−.103 (.110)	−.035 (.111)	−.080 (.079)	−.049 (.078)	.066 (.080)	.082 (.081)	.023 (.101)	.038 (.105)
Depression	.017 (.122)	.249 (.127)	.083 (.082)	.154 (.093)	.037 (.095)	.080 (.110)	−.104 (.107)	−.021 (.131)
*R* ^2^ (change)	.411*	.511*	.232*	.262*	.126*	.145*	.091	.104

Note

Table displays standardized regression coefficients (*SE*s in parentheses). NA = negative affect; PA = positive affect. *N* = 412.

* *p* < .05.

**Table 4:** Latent Change Regression Models: Predicting Change in Child Behavior and Parental Well‐Being From Daily Autonomy Support, Daily Parental Need Fulfillment, and Daily Child Well‐Being

Predictor	Emotional problems		Hyperactivity/inattention		Prosocial behavior		Vitality		Stress	
	Model 1	Model 2	Model 1	Model 2	Model 1	Model 2	Model 1	Model 2	Model 1	Model 2
Baseline level	−.435 (.064)*	−.696 (.078)*	−.396 (.062)*	−.424 (.066)*	−.346 (.071)*	−.433 (.087)*	−.513 (.042)*	−.636 (.049)*	−.622 (.036)*	−.772 (.049)*
Autonomy support	−.017 (.066)	.052 (.066)	−.159 (.090)	.013 (.093)	.043 (.083)	−.081 (.099)	.141 (.054)*	.012 (.055)	−.062 (.043)	.015 (.049)
Parental need satisfaction	—	.053 (.071)	—	−.042 (.085)	—	.092 (.098)	—	.304 (.059)*	—	−.138 (.059)*
Parental need frustration	—	.010 (.081)	—	−.010 (.102)	—	−.211 (.103)*	—	.023 (.068)	—	.264 (.061)*
Child PA	—	−.249 (.098)*	—	−.238 (.104)*	—	.312 (.147)*	—	.164 (.077)*	—	−.083 (.066)
Child NA	—	.389 (.108)*	—	.004 (.096)	—	.196 (.124)	—	−.080 (.069)	—	−.035 (.065)
Covariates										
Corona‐related worries	.161 (.059)*	.117 (.061)	.008 (.066)	.011 (.067)	−.007 (.083)	.016 (.085)	−.104 (.049)*	−.103 (.049)*	.195 (.044)*	.187 (.048)*
Homeschooling	.043 (.062)	.010 (.064)	.183 (.075)*	.157 (.073)*	−.054 (.083)	−.045 (.091)	−.049 (.057)	−.025 (.058)	.018 (.048)	.013 (.053)
Time spent with child	.085 (.071)	.087 (.068)	−.032 (.074)	−.006 (.074)	−.009 (.077)	−.049 (.077)	.070 (.052)	.045 (.051)	−.024 (.039)	−.018 (.042)
Parent gender	−.044 (.042)	−.068 (.043)	.012 (.060)	.016 (.060)	.012 (.066)	.004 (.069)	.008 (.036)	.011 (.036)	−.069 (.030)*	−.066 (.033)*
Target child's gender	.016 (.053)	.005 (.054)	.136 (.069)*	.143 (.067)*	−.137 (.069)*	−.120 (.073)	−.054 (.042)	−.057 (.043)	.061 (.037)	.055 (.041)
Target child’s age	−.133 (.061)*	−.124 (.067)	−.256 (.070)*	−.299 (.067)*	−.065 (.080)	−.029 (.081)	.115 (.048)*	.097 (.051)	−.182 (.041)*	−.185 (.045)*
Number of children in household	.009 (.051)	.009 (.055)	−.001 (.065)	−.010 (.067)	−.118 (.067)	−.090 (.071)	−.084 (.046)	−.089 (.048)	.128 (.043)*	.112 (.048)*
Parent employment: work away from home	−.027 (.054)	.032 (.054)	.067 (.063)	.098 (.064)	−.089 (.071)	−.121 (.072)	−.031 (.043)	−.062 (.046)	−.033 (.040)	−.009 (.044)
Parent employment: no employment	−.110 (.045)*	−.083 (.049)	−.013 (.063)	.006 (.061)	.092 (.066)	.078 (.067)	.054 (.046)	.035 (.043)	−.037 (.040)	−.028 (.041)
Expected change in income	.017 (.062)	−.007 (.066)	−.055 (.060)	−.055 (.062)	.189 (.062)*	.186 (.067)*	.131 (.050)*	.126 (.050)*	−.054 (.036)	−.030 (.038)
Days end of holidays to post assessment	.007 (.049)	.002 (.047)	−.091 (.050)	−.099 (.049)*	.105 (.062)	.123 (.069)	.003 (.047)	.002 (.044)	−.016 (.035)	−.027 (.033)
Loneliness	.056 (.065)	−.008 (.064)	−.079 (.070)	−.102 (.069)	−.001 (.082)	.059 (.082)	−.066 (.051)	−.013 (.053)	.080 (.044)	.020 (.049)
Depression	.118 (.070)	−.021 (.077)	.160 (.076)*	.061 (.084)	−.204 (.083)*	−.055 (.103)	−.119 (.063)	.006 (.074)	.163 (.061)*	.076 (.073)
*R* ^2^ (change)	.308*	.363*	.278*	.299*	.258*	.280*	.337*	.347*	.556*	.503*

Note.

Table displays standardized regression coefficients (*SE*s in parentheses). NA = negative affect; PA = positive affect. *N* = 412.

* *p* < .05.

To examine the unique effects of parental need satisfaction and frustration and daily child well‐being on change in adjustment measures above and beyond autonomy‐supportive parenting, we set up latent change models that included all these five predictors simultaneously. Results (Model 2; Tables 3 and 4) showed that only the effect of autonomy‐supportive behavior on cohesion remained statistically significant, β = .306, *p* = .025.

**Figure 1:**
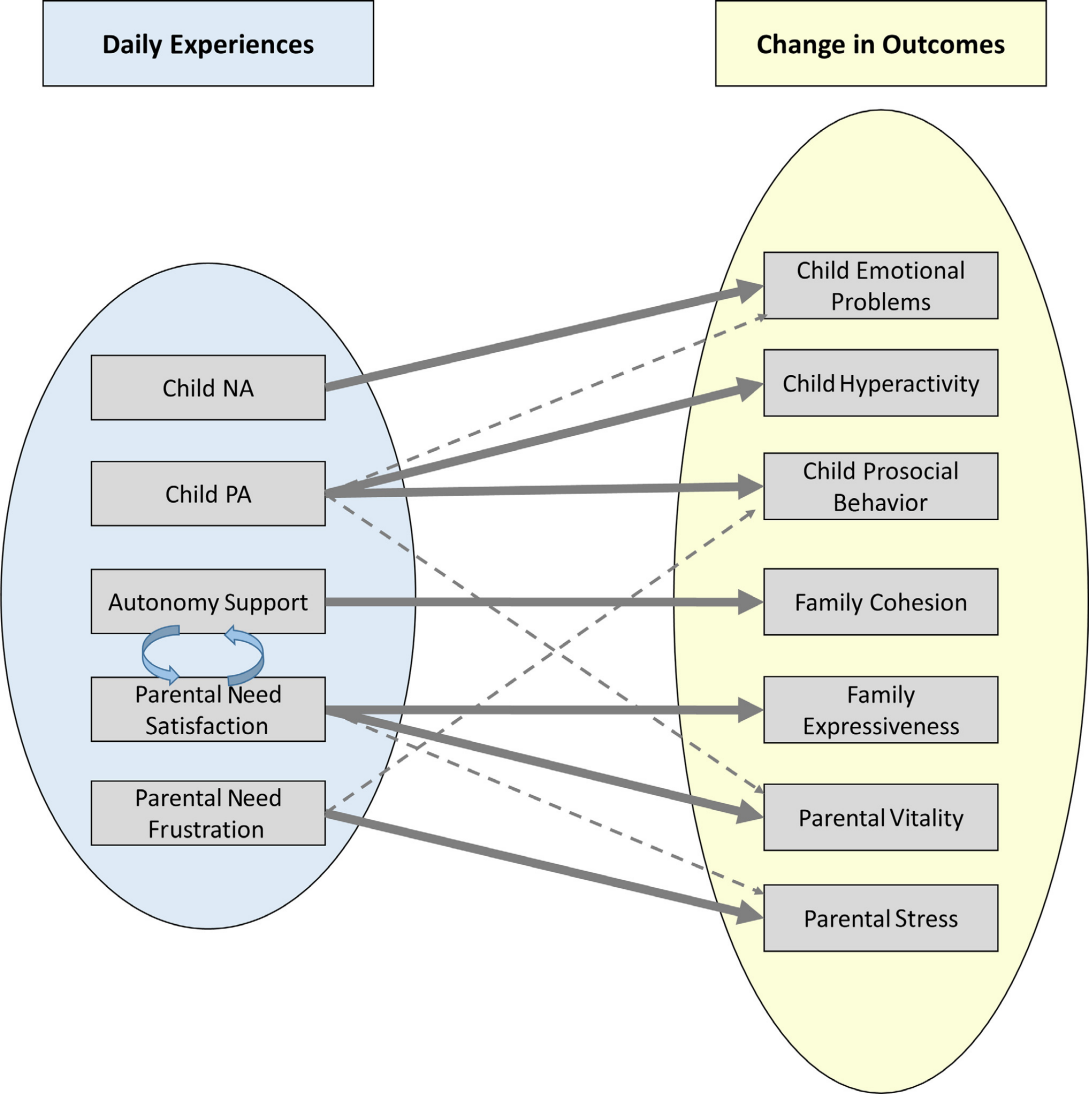
Schematic summary of the main findings. Associations in the left part (curved blue arrows) represent across‐day associations. Dashed gray arrows represent comparatively smaller effects. NA = negative affect; PA = positive affect.

Partially supporting our fourth research hypothesis, the findings for child well‐being predicting change in child behavior revealed that higher average child PA was associated with a decrease in emotional problems, β = −.249, *p* = .011, and hyperactivity, β = −.238, *p* = .022, as well as an increase in this child’s prosocial behavior, β = .312, *p* = .034. Higher average child NA was only significantly associated with an increase in child emotional problems, β = .389, *p* < .001, but not with the other two dimensions of child behavior, *p* > .114. Furthermore, and in line with our fourth research hypothesis, parental need satisfaction (frustration) uniquely predicted change in vitality (stress), β = .304, *p* < .001 (β = .264, *p* < .001). In addition to these predicted associations, there were some associations between daily experiences and change in the examined variables that had not been postulated a priori: Parental need satisfaction had unique associations with change in expressiveness, β = .201, *p* = .031, and parental perceived stress, β = −.138, *p* = .020. Need frustration had a unique association with change in children’s prosocial behavior, β = −.211, *p* = .040. Finally, participants who reported higher average child PA showed an increase in vitality, β = .164, *p* = .033. A conceptual summary of the study’s findings is depicted in Figure 1.

### Exploratory Analyses

Since the questionnaires in the post assessments referred to the previous week, and therefore to the last diary week, associations between daily assessments and retrospective assessments might have been inflated. We therefore removed the last week of the daily diary period and reran the latent change regression models: the effect of parental need satisfaction on change in perceived stress in Model 2 became smaller and was no longer statistically significant, β = −.107, *p* = .069. The pattern of all other effects remained unchanged.

Lastly, we explored if the associations between daily parental need fulfillment and daily autonomy‐supportive parenting, as well as between daily autonomy‐supportive parenting and daily child well‐being would differ depending on the target child’s age, child’s gender, or parent’s gender. Only two of the 12 cross‐level interaction effects were statistically significant, indicating that the effect of parental need satisfaction on daily autonomy‐supportive parenting was smaller for parents of older target children, *b* = −.023, *p* = .010, and somewhat stronger for fathers than for mothers, *b* = .160, *p* = .047. The other moderation effects were not statistically significant, *p* > .060 for all.

## Discussion

The measures implemented to slow down the spread of the Coronavirus in early 2020 have potentially had a large impact on school children, parents, and their families. To better understand parents’ and children’s adaptation to this critical event, we conducted a daily diary study embedded in pre–post longitudinal design and asked parents of school children repeatedly about their own experiences, their assessments of their child’s feelings and behaviors, and their perceptions of the family environment. Findings showed that—across 3 weeks of active counter‐Corona measures in Germany—there were no major average changes in parents’ well‐being, and parent‐rated child behavior and family environment. There were, however, meaningful interindividual differences in these adjustment measures, suggesting that individuals differed in the degree to which they adapted to this disturbance in their environment, which may suggest that families have been at different time points of the adjustment process to this stressful event (see Smyth et al., 2018). To better understand these differences between families, we targeted daily autonomy‐supportive parenting, daily parental need fulfillment, and daily child well‐being as potential predictors of these changes. Using intensive longitudinal data, we also examined the dynamic interplay of these variables.

### Autonomy Support: Short‐Term Effects on Children’s and Parents’ Adjustment

Findings from the 3‐week daily diary suggested that autonomy‐supportive parenting is positively associated with better child well‐being and higher parental need fulfillment (higher need satisfaction, lower need frustration) on the same day. These findings are in line with previous research emphasizing the important role of autonomy‐supportive parenting for child well‐being in cross‐sectional (Vasquez et al., 2016) and longitudinal studies (Joussemet et al., 2005). Notably, while prior studies have primarily examined same‐day within‐person associations among these constructs (van der Kaap‐Deeder et al., 2017), the present work also examined potential across‐day effects.

Our findings supported the prediction that autonomy‐supportive parenting and parental need satisfaction would be reciprocally related across time: On the one hand, need satisfaction was associated with more autonomy‐supportive parenting on the next day. This finding dovetails with previous research that has interpreted within‐day associations between these two constructs as indicating that need satisfaction provides the necessary resources to engage in autonomy‐supportive parenting (Dieleman et al., 2019). Going beyond prior work, our findings suggest that the energy fueling effect of need satisfaction reaches across days. This longitudinal effect further strengthens the argument that need satisfaction might in fact be associated with subsequent changes in parental behavior.

On the other hand, our findings also revealed a reverse effect from autonomy‐supportive parenting to higher need satisfaction on the next day. This finding is in line with the suspected, but previously untested, reciprocal relation among autonomy‐supportive parenting and parental need satisfaction (Mabbe et al., 2018). The exact mechanism by which autonomy‐supportive parenting might affect parental need fulfillment was not targeted in the present research and needs to be explored in future studies. Potentially, just like hurting other people’s needs has been suggested to be detrimental to one’s own needs (Legate, DeHaan, Weinstein, & Ryan, 2013), supporting others’ needs via autonomy‐supportive parenting might in itself be inherently satisfying and thereby directly facilitate one’s own basic psychological needs (Deci et al., 2006). Alternatively, more favorable child behavior as a consequence of autonomy‐supportive parenting could feed back into subsequent parental need satisfaction. That is, a child’s behavior on days when his or her parent provided more autonomy support might lead to higher subsequent need satisfaction on part of the parent: Children might be more engaged with their own daily activities, less prone to repeatedly ask their parents for assistance or directions, which could free up time for their parents they can use to fulfill their own needs in turn. In interpreting our findings, it needs to be considered that, while the across‐day effects were small in magnitude (β < .045), these effects can accumulate over time, providing the potential for sizable cumulative effects over longer time periods.

Contrary to our expectations, there were no effects of autonomy‐supportive parenting on next‐day child well‐being. As the same‐day associations were statistically meaningful, this pattern of findings suggests that the effect of autonomy support on child well‐being might be more direct and occur on the same day, but have no effects on next‐day well‐being after controlling for today’s well‐being. To examine the possibility of potentially stronger within‐day effects, future research should consider using experience sampling studies with multiple assessments per day to examine the lead–lag association among these constructs within days.

Our exploratory analyses on age moderation further suggest that the same‐day effect of parental need satisfaction on autonomy‐supportive parenting might be larger for younger children which suggests that older children might receive autonomy support more independently from their parent’s own daily experiences. We note that this finding resulted from exploratory analyses which need to be interpreted particularly carefully.

### Daily Experiences as Predictors of Change in Adjustment

Daily autonomy‐supportive parenting was associated with an increase in positive emotional family climate (specifically: family cohesion) and in parent’s own vitality. That is, there is some evidence that autonomy‐supportive parenting might have positive short‐term consequences on psychological adjustment (across 3 weeks) during the COVID‐19 pandemic. We hasten to add, however, that we found no evidence for the effects of autonomy‐supportive parenting on change in parental stress, child behavior, or the other dimensions of perceived family environment (expressiveness, organization, control). The potentially positive effects of autonomy‐supportive parenting, therefore, seem to be outcome specific and do not generalize across all outcomes tested in the present work.

Given the mutual interplay among autonomy‐supportive parenting, parental need fulfillment, and child well‐being that has been established in prior research (van der Kaap‐Deeder et al., 2017, 2019) and that has also been found on a day‐to‐day level in the present work, we took a more fine‐grained look at the effects and tested if the positive effects of autonomy‐supportive parenting would remain once we controlled for average parental need fulfillment and child well‐being. Finding showed that only the effect on change in family cohesion remained statistically significant after controlling for these daily experiences. In addition, daily child well‐being was related to changes in child behavior: Specifically, children who (according to their parents) experienced more PA across the 3 weeks were reported as decreasing in hyperactivity and emotional problems, as well as increasing in their prosocial behavior. Furthermore, daily child NA was related to an increase in emotional problems. This suggests that children’s daily affective experiences might drive longitudinal change in how their behavior is perceived.

Parents who reported higher satisfaction of their needs for autonomy, competence, and relatedness reported more increases in vitality and decreases in stress experiences. Need frustration was only associated with changes in perceived stress, but not with changes in vitality. This pattern of findings is largely consistent with previous research that has tied need satisfaction primarily to well‐being outcomes, and need frustration primarily to ill‐being outcomes (Chen et al., 2015; Schmidt, Neubauer, Dirk, & Schmiedek, 2020), highlighting that need satisfaction and need frustration are more than opposite poles of a single need fulfillment dimension (Neubauer & Voss, 2018) and that these two dimensions potentially differ in both their antecedents and their outcomes (Vansteenkiste & Ryan, 2013).

Together, these findings indicate that autonomy‐supportive parenting might have beneficial downstream effects on adjustment measures on the child, parent, and family level: Autonomy‐supportive parenting is associated with child well‐being on a daily level, which, in turn, is associated with longitudinal change in internalizing behavior (emotional problem), externalizing behavior (hyperactivity), and prosocial behavior. When providing autonomy support to their child, parent’s need fulfillment increases on the next day, and parental need fulfillment accounts for longitudinal change in parental well‐being across 3 weeks. Finally, autonomy‐supportive parenting is directly associated with an increase in family cohesion as perceived by the parent. These findings suggest that potentially minor parenting behavior choices could lead longitudinally to better or worse adjustment for families with school children during the COVID‐19 pandemic in Germany. The effects of daily experiences on change in outcome measures reached sizes that are typically considered small to medium (see Tables 3 and 4 for standardized regression coefficients), suggesting that these effects may provide practical utility as well, for example, by means of interventions fostering autonomy‐supportive parenting (Joussemet, Mageau, & Koestner, 2014). Notably, even though autonomy‐supportive parental behavior had no unique effects on change in parental well‐being or child behavior after controlling for daily parental need fulfillment, autonomy‐supportive parenting might have indirect effects on these outcomes via parental need satisfaction and daily child well‐being. This suggests that interventions targeting autonomy‐supportive parenting might improve family cohesion directly, and, for example, parental well‐being indirectly, mediated via parental need satisfaction. Similarly, improving parental need satisfaction might be associated with positive changes in family climate indirectly via autonomy‐supportive parenting. The implementation of such interventions on a day‐to‐day level (Schmiedek & Neubauer, 2020) could further aid in testing the potential causal effects of autonomy‐supportive parenting on child well‐being and parental need fulfillment.

### Limitations

A number of limitations has to be considered when interpreting the findings from this study. First, we only assessed one aspect of autonomy‐supportive parenting: choice within limits (Mageau et al., 2015). Rationales for demands or acknowledgments of feelings have also been described as important dimensions of autonomy‐supportive parenting behavior. Similarly, controlling parenting as well as provision of structure and parental involvement (Ryan & Deci, 2017) were also not examined in this work, limiting the implications of the results for parenting behavior only to the provision of choice within limits. Specifically, parental involvement might be related to expressiveness, which was, contrary to our expectations, not related to autonomy‐supportive parenting. Although more research on this topic is clearly needed, our findings suggest that a focused assessment of autonomy‐supportive parenting as choice within limits revealed beneficial consequences on some adjustment outcomes. Second, we used a questionnaire to assess parents’ perceptions of the family environment that had only been used in adolescents thus far (Roth, 2002). Our psychometric analysis of this scale revealed a theoretically plausible factor structure, yet the interpretation of this structure awaits further validation. Third, all reports were obtained from the parent perspective. The observed associations may therefore have been affected by a perceiver effect, with a parent’s general positive self‐view on one day leading to an overly positive perception of their parenting style, as well as the perception of better own and child well‐being. While we controlled for some variables on the person and day level that might remove such common influences (depression, loneliness, and daily worries), other confounding common causes cannot be excluded here. In particular the assessments of child well‐being might therefore be a nonperfect measure of the children’s true affective state on the respective day. Because we intended to include data from all school‐aged children (a broad age range from 6 to 19 years), children’s self‐reports were difficult to obtain since the items to capture affect would likely not be comprehensible for the youngest children in the sample. Future studies might consider including measures from multiple sources (e.g., both parents) or from children directly, for example, via video calls. Fourth, it needs to be considered that the present sample is positively selected in terms of educational background and financial situation. Potentially, the effect of autonomy‐supportive parenting might be different in samples of more disadvantaged socioeconomic backgrounds. Furthermore, the study was conducted in Germany and the extent to which similar findings would be observed in different countries cannot be determined in this work. Given SDT’s universality claim and previous studies that have reported comparable effects of autonomy‐supportive parenting across countries (Lekes et al., 2010), it could be expected that these effects can generalize to other countries as well, yet this needs to be determined in further research. In addition, parents participating in this study were predominantly female, which needs to be considered as a factor potentially limiting the generalizability of our findings. In a similar vein, while targeting the dynamics of daily experiences and change in adjustment during this pandemic allows for examining this interplay in this specific situation, it is unclear to what extent these findings can be generalized to other contexts (i.e., to before or after the pandemic). The specific setting (with potentially increased stress and more frequent parent‐child interactions) might have an impact on both the day‐to‐day dynamics and the interplay between daily dynamics and longitudinal change. Fifth, compliance rates were below 80% in the daily diary part of this study. Although a higher compliance rate would have been desirable, we consider this compliance rate adequate considering that this was a 3‐week daily diary study without direct remuneration in a very demanding time period (during the COVID‐19 pandemic).

### Conclusions

Our findings suggest that families with school children differ in their adaptation to the measures that have been enforced to slow down the spread of the Coronavirus in Germany. Daily experiences predict changes in this adaptation over a period of 3 weeks. Autonomy‐supportive parenting was associated with improved family cohesion across 3 weeks. Parents’ fulfillment of the basic psychological needs for autonomy, competence, and relatedness was associated with an increase in parents’ vitality and a decrease in their perceived stress. Parents’ perceptions of their children’s daily well‐being were related to more adaptive changes in (parent‐rated) child behavior. Findings also showed that daily autonomy‐supportive parenting was associated with parental need fulfillment and child well‐being. The dynamic interplay between autonomy‐supportive parenting and need satisfaction was reciprocal, suggesting a potential positive upward spiral among these constructs. In sum, autonomy‐supportive behavior might have positive downstream effects not only on the receiving child, but also on the social system (the family) and the support provider—also in challenging times as during the Corona crisis.

## Supporting information


**Table S1.** Comparing Drop‐Outs After Study Part 1 to Continuers
**Table S2.** Overview of Constructs Used in This Work
**Table S3.** Descriptive Statistics on the Person Level
**Table S4.** Results of the Exploratory Factor Analysis on the 17 Family Environment Items
**Table S5.** Model Fit Indices of the Individual Measurement Models in the Two Independent Samples
**Table S6.** Multilevel Model: Predicting Autonomy‐Supportive Parenting and Child Well‐Being
**Table S7.** Results From the Dynamic Structural Equation Model
**Table S8.** Standardized Factor Loadings of the Indicators
**Table S9.** Tests of Measurement Invariance
**Table S10.** Latent Difference Score Model: Intercorrelations of Latent Variables
**Appendix S1.** Factor Structure of the Measurement Scales
**Appendix S2.** Measurement Invariance
