# Supplementary material for: A Little Autonomy Support Goes a Long Way: Daily Autonomy‐Supportive Parenting, Child Well‐Being, Parental Need Fulfillment, and Change in Child, Family, and Parent Adjustment Across the Adaptation to the COVID‐19 Pandemic
Source: Child Dev. 2021 Jan 19;92(5):1679–97. doi: 10.1111/cdev.13515 (PMC8013550; doi:10.1111/cdev.13515)
Supplement: Supplementary file 1 — Table S1. Comparing Drop‐Outs After Study Part 1 to Continuers Table S2. Overview of Constructs Used in This Work Table S3. Descriptive Statistics on the Person Level Table S4. Results of the Exploratory Factor Analysis on the 17 Family Environment Items Table S5. Model Fit Indices of the Individual Measurement Models in the Two Independent Samples Table S6. Multilevel Model: Predicting Autonomy‐Supportive Parenting and Child Well‐Being Table S7. Results From the Dynamic Structural Equation Model Table S8. Standardized Factor Loadings of the Indicators Table S9. Tests of Measurement Invariance Table S10. Latent Difference Score Model: Intercorrelations of Latent Variables Appendix S1. Factor Structure of the Measurement Scales Appendix S2. Measurement Invariance [file CDEV-92-1679-s001.docx]

**Contents**

Table S1. Comparing Drop-Outs After Study Part 1 to Continuers.

Table S2. Overview of Constructs Used in This Work.

Table S3. Descriptive Statistics on the Person Level.

Table S4. Results of the Exploratory Factor Analysis on the 17 Family Environment Items.

Table S5. Model Fit Indices of the Individual Measurement Models in the Two Independent Samples.

Table S6. Multilevel Model: Predicting Autonomy-Supportive Parenting and Child Well-Being.

Table S7. Results From the Dynamic Structural Equation Model.

Table S8. Standardized Factor Loadings of the Indicators.

Table S9. Tests of Measurement Invariance.

Table S10. Latent Difference Score Model: Intercorrelations of Latent Variables.

Appendix S1. Factor Structure of the Measurement Scales.

Appendix S2. Measurement Invariance.

Table S1.

*Comparing Drop-Outs After Study Part 1 to Continuers.*

| Variable | Mean (*SD*) drop outs | Mean (*SD*) continuers | Difference test |
| --- | --- | --- | --- |
| Participant age | 43.11 (6.77) | 42.79 (6.12) | *t*(818.77) = 0.75  *p* = .454 |
| Child age | 9.92 (2.90) | 9.74 (2.81) | *t*(857.55) = 0.95  *p* = .343 |
| Expected change in income | -0.48 (0.98) | -0.45 (0.99) | *t*(794.90) = -0.55  *p* = .584 |
| Number of children in household | 2.00 (0.78) | 2.01 (0.88) | *t*(919.64) = -0.32  *p* = .752 |
| SDQ: Emotional Problems | 1.89 (2.01) | 2.14 (2.21) | *t*(900.77) = -1.81  *p* = .070 |
| SDQ: Hyperactivity / Inattention | 4.01 (2.62) | 4.19 (2.79) | *t*(888.50) = -0.99  *p* = .319 |
| SDQ: Prosocial Behavior | 7.34 (2.12) | 7.23 (2.20) | *t*(875.97) = 0.80  *p* = .425 |
| Stress | 3.84 (1.28) | 3.86 (1.30) | *t*(875.49) = -0.26  *p* = .796 |
| Vitality | 4.29 (1.38) | 4.13 (1.36) | *t*(649.93) = 1.70  *p* = .091 |
| Family Environment: Cohesion | 3.76 (0.72) | 3.80 (0.67) | *t*(697.36) = -1.01  *p* = .311 |
| Family Environment: Expressiveness | 4.44 (0.72) | 4.52 (0.66) | *t*(679.60) = -1.65  *p* = .100 |
| Family Environment: Organization | 3.55 (0.85) | 3.49 (0.86) | *t*(743.13) = 1.04  *p* = .298 |
| Family Environment: Control | 3.12 (0.76) | 3.13 (0.71) | *t*(697.53) = -0.23  *p* = .817 |
| Depression | 15.48 (9.98) | 16.11 (10.09) | *t*(750.43) = -0.92  *p* = .357 |
| Loneliness | 2.70 (1.12) | 2.67 (1.17) | *t*(667.23) = 0.46  *p* = .644 |
|  | Frequencies drop outs | Frequencies continuers |  |
| Parent gender (female / male /  non binary / no response) | 349 / 58 / 0 / 1 | 489 / 71 / 0 / 1 | χ²(1) = 0.38  *p* = .539 |
| Child gender (female / male /  non binary / no response) | 192 / 210 / 3 / 2 | 268 / 290 / 0 / 3 | χ²(1) < 0.01  *p* = .987 |
| Relationship status (single /  in permanent relationship, separate households /  in permanent relationship, shared household /  married / separated or divorced / widowed | 25 / 14 / 52 / 265 / 50 / 2 | 28 / 14 / 64 / 382 / 64 / 9 | χ²(4) = 2.09  *p* = .720 |
| Income (less than 500€ /  500€ to < 1000€ / 1000€ to < 1500€ /  1500€ to < 2000€ / 2000€ to < 2500€ / 2500€ to < 3000€ / 3000€ to < 3500€ / 3500€ to < 4000€ / 4000€ or more /  I prefer to not answer this question.) | 1 / 2 / 14 / 18 / 35 / 39 / 37 / 55 / 169 / 31 | 0 / 6 / 11 / 28 / 33 / 50 / 44 / 72 / 277 / 37 | χ²(7) = 8.78  *p* = .269 |
| School type (primary school /  lower secondary school (German:  Mittelschule / Hauptschule) /  intermediate secondary school (German: (Werk-)Realschule) / vocational school, college /  grammar school / academic tier  secondary school (German: Gymnasium) /  comprehensive school (German:  Gesamtschule) /  other school type) | 243 / 1 / 10 / 0 / 105 / 25 / 22 | 367 / 2 / 7 / 0 / 135 / 28 / 22 | χ²(4) = 5.19  *p* = .269 |
| Employment (no employment / working from home / working away from home) | 43 / 251 / 104 | 50 / 361 / 144 | χ²(2) = 0.91  *p* = .635 |

*Note.* For all χ²-tests categories with five or fewer observation in the drop-out and / or continuer group were removed. Results did not differ when including these low count categories.

Table S2.

*Overview of Constructs Used in This Work..*

| Construct | Assessed at baseline | Assessed at post assessments | Assessed in the daily diary |
| --- | --- | --- | --- |
| Child behavior:   - Emotional problems - Hyperactivity / inattention - Prosocial behavior | Yes | Yes | No |
| Family environment   - Cohesion - Expressiveness - Organization - Control | Yes | Yes | No |
| Perceived Stress | Yes | Yes | No |
| Vitality | Yes | Yes | No |
| Autonomy-supportive parenting | No | No | Yes |
| Child well-being   - Positive affect - Negative affect | No | No | Yes |
| Parental need fulfillment   - Need satisfaction - Need frustration | No | No | Yes |

Table S3.

*Descriptive Statistics on the Person Level.*

|  |  | Correlations | | | | | | | | | | | | | |
| --- | --- | --- | --- | --- | --- | --- | --- | --- | --- | --- | --- | --- | --- | --- | --- |
|  |  | 1 | 2 | 3 | 4 | 5 | 6 | 7 | 8 | 9 | 10 | 11 | 12 | 13 | 14 |
| 1 | Autonomy support |  | .40 | -.34 | .43 | -.34 | .24  .34 | .14  .11 | .07  .13 | -.10  -.06 | -.17  -.19 | -.30  -.30 | .16  .16 | .25  .28 | -.25  -.26 |
| 2 | Need satisfaction |  |  | -.42 | .56 | -.38 | .30  .41 | .21  .25 | .25  .26 | .18  .19 | -.25  -.27 | -.30  -.30 | .23  .27 | .50  .53 | -.45  -.46 |
| 3 | Need frustration |  |  |  | -.50 | .64 | -.31  -.39 | -.27  -.28 | -.32  -.33 | -.06  -.06 | .35  .38 | .27  .25 | -.23  -.31 | -.37  -.37 | .49  .55 |
| 4 | Child PA |  |  |  |  | -.65 | .29  .37 | .20  .18 | .24  .24 | .16  .21 | -.40  -.47 | -.37  -.41 | .38  .44 | .43  .47 | -.44  -.39 |
| 5 | Child NA |  |  |  |  |  | -.22  -.27 | -.21  -.18 | -.23  -.26 | -.06  -.10 | .51  .58 | .31  .30 | -.24  -.28 | -.33  -.37 | .45  .43 |
| 6 | Cohesion |  |  |  |  |  | .71 | .35  .35 | .25  .28 | .05  -.03 | -.06  -.16 | -.19  -.25 | .31  .28 | .25  .22 | -.21  -.28 |
| 7 | Expressiveness |  |  |  |  |  | .36 | .63 | .23  .21 | .01  .04 | -.07  -.14 | -.09  -.08 | .13  .16 | .16  .11 | -.14  -.14 |
| 8 | Organization |  |  |  |  |  | .22 | .20 | .80 | .37  .42 | -.19  -.17 | -.21  -.17 | .21  .21 | .28  .19 | -.25  -.23 |
| 9 | Control |  |  |  |  |  | .00 | .02 | .34 | .77 | -.04  -.04 | -.02  -.01 | .12  .12 | .20  .16 | -.10  .00 |
| 10 | Emotional problems |  |  |  |  |  | -.14 | -.05 | -.17 | -.13 | .63 | .34  .28 | -.17  -.21 | -.28  -.30 | .35  .31 |
| 11 | Hyperactivity |  |  |  |  |  | -.23 | -.02 | -.20 | -.05 | .31 | .76 | -.41  -.37 | -.37  -.37 | .35  .30 |
| 12 | Prosocial behavior |  |  |  |  |  | .27 | .11 | .24 | .13 | -.14 | -.33 | .69 | .27  .23 | -.28  -.22 |
| 13 | Vitality |  |  |  |  |  | .23 | .09 | .21 | .16 | -.33 | -.34 | .30 | .64 | -.62  -.52 |
| 14 | Stress |  |  |  |  |  | -.25 | -.11 | -.23 | -.11 | .32 | .32 | -.30 | -.47 | .62 |
|  | Mean (*SD*) | 5.43 (.87) | 4.40 (.89) | 2.18 (.86) | 5.15 (.85) | 2.09 (.82) | 3.79 (.69)  3.83 (.69) | 4.49 (.68)  4.50 (.69) | 3.51 (.86)  3.45 (.88) | 3.13 (.73)  3.08 (.75) | 2.04 (2.14)  2.01 (2.24) | 4.11 (2.72)  4.12 (2.72) | 7.27 (2.16)  7.49 (2.12) | 4.19 (1.36)  4.22 (1.42) | 3.86 (1.29)  3.74 (1.26) |
|  | Number of valid observations | 559 | 554 | 554 | 557 | 557 | 908  467 | 908  467 | 908  467 | 908  467 | 960  469 | 960  469 | 960  469 | 879  465 | 929  467 |

*Note.* Table depicts means, standard deviations, and correlations of the study variables on the person level. Within each cell, the first correlation / mean / standard deviation refers to the estimates for the baseline assessment (t1), the second entry relates to the results from the post assessment (t2). *SD =* Standard deviation.

Table S4.

*Results of the Exploratory Factor Analysis on the 17 Family Environment Items.*

|  | Loadings | | | | h2 |
| --- | --- | --- | --- | --- | --- |
| Item | Organization | Control | Expressiveness | Cohesion |  |
| 1: Each member of our family has the same rights when decisions are made. | -.01 | -.07 | -.05 | **.81** | .65 |
| 4^a^: In our family, it is not well liked if someone says he or she is not happy with something. | .04 | .00 | **.53** | .06 | .32 |
| 5^a^: In our family, we are rather reluctant about openly voicing our opinion. | .05 | -.03 | **.82** | -.08 | .66 |
| 11: In our family, it is ok for everyone to stand up for their own interests. | -.23 | .15 | .37 | **.30** | .27 |
| 14: In our family, everyone can pursue their own interests without the others being angry. | -.04 | .02 | .31 | **.36** | .27 |
| 17: At home each voice counts equally when something has to be decided that concerns the entire family. | .06 | -.02 | -.01 | **.75** | .58 |
| 20^a^: In our family, feelings are rather not shown. | .01 | -.04 | **.59** | .01 | .35 |
| 23: In our family, members address each other's worries. | .06 | .13 | .37 | **.35** | .38 |
| 6^a^: Things are often chaotic in our family because nothing is really planned. | **.77** | -.03 | .00 | -.01 | .57 |
| 10^a^: We have to improvise a lot because nothing is really planned. | **.68** | .11 | -.01 | .00 | .53 |
| 19^a^: We do everything last minute. | **.71** | .03 | .07 | .02 | .55 |
| 29^a^: It is sometimes difficult in our family to make sure that all are looked after. | **.40** | -.10 | .11 | .12 | .22 |
| 7: The rules in our family are quite strictly observed. | .10 | **.62** | .00 | -.05 | .45 |
| 9: We have certain rules as to how things are done. | .01 | **.70** | -.02 | -.06 | .50 |
| 12: At home, we have rather fixed rules as to what is allowed and what isn't. | -.09 | **.71** | -.01 | -.07 | .49 |
| 15: In our family, duties are clearly assigned and each member knows what he or she has to do. | .14 | **.53** | .01 | .13 | .36 |
| 24: In our family, we highly value timeliness. | .24 | **.36** | -.08 | .19 | .28 |
|  | Correlations | | | |  |
| Control | .39 |  |  |  |  |
| Expressiveness | .24 | .03 |  |  |  |
| Cohesion | .20 | -.11 | .29 |  |  |

*Note.* Table depicts standardized factor loadings and communalities (h2) of the 17 items and correlations of the four extracted factors. Loadings in bold represent the items’ loadings on their designated factor. Note that factor assignment was based on both statistical and theoretical considerations. The numbers preceding the items refer to the position of these items in the original questionnaire used by Roth (2002).

^a^ Item was recoded prior to the analyses

Table S5.

*Model Fit Indices of the Individual Measurement Models in the Two Independent Samples.*

| Scale | χ² | c | df | CFI | RMSEA^+^ | SRMR | BIC |
| --- | --- | --- | --- | --- | --- | --- | --- |
|  | Sample 1 | | | | | | |
| Family environment | 408.918 | 1.0849 | 113 | .831 | .076  [.068, .083] | .080 | 21327.635 |
| Family environment (cov) | 308.027 | 1.0910 | 112 | .888 | .062  [.054, .070] | .067 | 21226.208 |
| SDQ | 400.828 | 1.0651 | 87 | .841 | .087  [.078, .095] | .067 | 11797.861 |
| SDQ (cov) | 237.047 | 1.0896 | 86 | .923 | .060  [.051, .070] | .061 | 11635.381 |
| Vitality | 62.456 | 1.4918 | 14 | .962 | .088  [.067, .111] | .030 | 9980.939 |
| Vitality (cov) | 38.327 | 1.4023 | 13 | .980 | .066  [.043, .091] | .022 | 9947.612 |
| Stress | 184.868 | 1.1092 | 35 | .898 | .096  [.082, .109] | .052 | 17553.909 |
| Stress (cov) | 156.843 | 1.0999 | 34 | .916 | .088  [.074, .102] | .048 | 17527.511 |
|  | Sample 2 | | | | | | |
| Family environment (cov) | 270.691 | 1.0752 | 112 | .900 | .056  [.048, .065] | .065 | 20000.103 |
| SDQ (cov) | 199.403 | 1.0771 | 86 | .935 | .052  [.042, .062] | .059 | 11583.676 |
| Vitality (cov) | 68.160 | 1.2896 | 13 | .963 | .099  [.076, .123] | .028 | 9417.005 |
| Stress (cov) | 214.003 | 1.1954 | 34 | .871 | .107  [.094, .121] | .055 | 17244.941 |

*Note.* c = scaling factor; df = degrees of freedom; CFI = comparative fit index; RMSEA = root mean square error of approximation; SRMR = standardized root mean square residual; BIC = Bayesian Information Criterion. (cov) indicate models in which a residual covariance (based on modification indices in Sample 1) was freely estimated.

^+^: 90% confidence interval in brackets.

Table S6.

*Multilevel Model: Predicting Autonomy-Supportive Parenting and Child Well-Being.*

| Predictor | Outcome:  Autonomy-supportive parenting | Outcome:  Child positive affect | Outcome:  Child negative affect |
| --- | --- | --- | --- |
|  | Fixed effects | | |
| Intercept | 5.580* (.057) | 5.122* (.049) | 2.210* (.050) |
| Within effects |  |  |  |
| Parental need satisfaction^a^ | .137* (.025) | --- | --- |
| Parental need frustration^a^ | -.191* (.024) | --- | --- |
| Autonomy-supportive parenting ^a^ | --- | .277* (.014) | -.176* (.014) |
| Between effects |  |  |  |
| Parental need satisfaction^b^ | .246* (.046) | --- | --- |
| Parental need frustrations^b^ | -.178* (.051) | --- | --- |
| Autonomy-supportive parenting^b^ | --- | .314* (.037) | -.185* (.037) |
| Time-varying covariates |  |  |  |
| Weekend^c^ | .074* (.028) | .090* (.022) | -.021 (.021) |
| Corona worries^a^ | -.006 (.005) | -.066* (.008) | .087* (.008) |
| Homeschooling^a^ | -.468* (.030) | -.048* (.024) | -.001 (.023) |
| Time spent with child^a^ | -.006 (.005) | .026* (.004) | .000 (.004) |
| Person-level covariates |  |  |  |
| Corona worries^b^ | .021 (.028) | -.053* (.023) | .116* (.023) |
| Homeschooling^b^ | -.432* (.147) | -.230 (.126) | .160 (.127) |
| Time spent with child^b^ | -.029* (.011) | .015 (.010) | .014 (.010) |
| Parent gender^d^ | -.070 (.108) | -.088 (.093) | .081 (.093) |
| Target child gender^d^ | -.183* (.067) | -.085 (.058) | -.094 (.058) |
| Target child age^e^ | .023 (.014) | -.045* (.012) | -.002 (.012) |
| Number of children in household^f^ | .070 (.043) | -.008 (.037) | -.018 (.037) |
| Parent employement: work away from home^g^ | -.149 (.077) | .145* (.066) | -.209* (.067) |
| Parent employment: no employment^g^ | -.061 (.121) | .194 (.103) | -.204* (.104) |
| Expected change in income | -.027 (.034) | -.016 (.029) | .015 (.029) |
| Loneliness^b^ | .013 (.033) | -.062* (.028) | .113* (.028) |
| Depression^b^ | -.003 (.004) | -.031* (.004) | .023* (.004) |
|  | Random effects (Variances) | | |
| Intercept | .453 | .336 | .357 |
| Parental need satisfaction | .092 | --- | --- |
| Parental need frustration | .084 | --- | --- |
| Autonomy-supportive parenting | --- | .036 | .035 |
| Level-1 Residual | .874 | .548 | .517 |

*Note.* Table displays unstandardized coefficients (standard errors for fixed effects in parentheses). Number of participants: 497; number of observations: 6,676 - 6,677

^a^time-varying predictor centered on the person-mean; ^b^person-level predictor centered on the grand mean; ^c^0= during the week, 1= weekend/holiday

^d^1= male, 0 = otherwise; ^e^Centered on 10 years; ^f^Centered on two children; ^g^dummy coded.

**p* < .05.

Table S7.

*Results From the Dynamic Structural Equation Model.*

|  | Estimate |
| --- | --- |
| Autoregressive effects |  |
| Autonomy supportive behavior | **.232 [.203, .261]** |
| Parental need satisfaction | **.216 [.185, .248]** |
| Parental need frustration | **.217 [.186, .248]** |
| Child positive affect | **.125 [.090, .160]** |
| Child negative affect | **.164 [.130, .200]** |
| Cross-regressive effects |  |
| Autonomy supportive behavior 🡪 Parental need satisfaction | **.030 [.008, .051]** |
| Autonomy supportive behavior 🡪 Parental need frustration | -.020 [-.042, .002] |
| Autonomy supportive behavior 🡪 Child positive affect | .019 [-.006, .045] |
| Autonomy supportive behavior 🡪 Child negative affect | -.009 [-.032, .015] |
| Parental need satisfaction 🡪 Autonomy supportive behavior | **.054 [.008, .100]** |
| Parental need satisfaction 🡪 Parental need frustration | **-.041 [-.077, -.007]** |
| Parental need satisfaction 🡪 Child positive affect | .012 [-.026, .049] |
| Parental need satisfaction 🡪 Child negative affect | .010 [-.023, .044] |
| Parental need frustration 🡪 Autonomy supportive behavior | .019 [-.021, .060] |
| Parental need frustration 🡪 Parental need satisfaction | .020 [-.011, .051] |
| Parental need frustration 🡪 Child positive affect | .035 [.000, .069] |
| Parental need frustration 🡪 Child negative affect | .021 [-.014, .056] |
| Child positive affect 🡪 Autonomy supportive behavior | .005 [-.040, .047] |
| Child positive affect 🡪 Parental need satisfaction | -.018 [-.048, .013] |
| Child positive affect 🡪 Parental need frustration | **.034 [.001, .067]** |
| Child positive affect 🡪 Child negative affect | -.008 [-.041, .023] |
| Child negative affect 🡪 Autonomy supportive behavior | -.028 [-.074, .020] |
| Child negative affect 🡪 Parental need satisfaction | -.011 [-.042, .021] |
| Child negative affect 🡪 Parental need frustration | .006 [-.030, .043] |
| Child negative affect 🡪 Child positive affect | **-.056 [-.093, -.018]** |
| Covariates |  |
| Weekend^a^ 🡪 Autonomy supportive behavior | -.040 [-.100, .018] |
| Weekend^a^ 🡪 Parental need satisfaction | .022 [-.018, .065] |
| Weekend^a^ 🡪 Parental need frustration | **-.119 [-.163, -.075]** |
| Weekend^a^ 🡪 Child positive affect | .034 [-.015, .083] |
| Weekend^a^ 🡪 Child negative affect | -.018 [-.063, .025] |
| Homeschooling^b^ 🡪 Autonomy supportive behavior | **-.329 [-.383, -.270]** |
| Homeschooling^b^🡪 Parental need satisfaction | **-.051 [-.091, -.009]** |
| Homeschooling^b^🡪 Parental need frustration | .020 [-.025, .063] |
| Homeschooling^b^🡪 Child positive affect | **-.119 [-.166, -.069]** |
| Homeschooling^b^🡪 Child negative affect | **.048 [.006, .091]** |
| Time spent with child 🡪 Autonomy supportive behavior | -.002 [-.012, .008] |
| Time spent with child 🡪 Parental need satisfaction | **.008 [.001, .015]** |
| Time spent with child 🡪 Parental need frustration | -.006 [-.013, .002] |
| Time spent with child 🡪 Child positive affect | **.025 [.017, .033]** |
| Time spent with child 🡪 Child negative affect | .000 [-.008, .008] |
| Corona worries 🡪 Autonomy supportive behavior | **-.034 [-.054, -.013]** |
| Corona worries 🡪 Parental need satisfaction | **-.058 [-.072, -.044]** |
| Corona worries 🡪 Parental need frustration | **.084 [.068, .099]** |
| Corona worries 🡪 Child positive affect | **-.072 [-.089, -.055]** |
| Corona worries 🡪 Child negative affect | **.085 [.070, .101]** |
| Random variances |  |
| Autonomy supportive behavior | **.553 [.472, .642]** |
| Parental need satisfaction | **.668 [.584, .767]** |
| Parental need frustration | **.589 [.515, .674]** |
| Child positive affect | **.558 [.488, .644]** |
| Child negative affect | **.534 [.466, .617]** |
| Autonomy supportive behavior 🡪 Parental need satisfaction | **.004 [.001, .010]** |
| Autonomy supportive behavior 🡪 Parental need frustration | **.002 [.001, .006]** |
| Autonomy supportive behavior 🡪 Child positive affect | **.005 [.001, .011]** |
| Autonomy supportive behavior 🡪 Child negative affect | **.008 [.003, .013]** |
| Parental need satisfaction 🡪 Autonomy supportive behavior | **.033 [.011, .060]** |
| Parental need satisfaction 🡪 Parental need frustration | **.021 [.011, .036]** |
| Parental need satisfaction 🡪 Child positive affect | **.016 [.006, .029]** |
| Parental need satisfaction 🡪 Child negative affect | **.007 [.001, .018]** |
| Parental need frustration 🡪 Autonomy supportive behavior | **.016 [.002, .035]** |
| Parental need frustration 🡪 Parental need satisfaction | **.010 [.003, .021]** |
| Parental need frustration 🡪 Child positive affect | **.010 [.002, .024]** |
| Parental need frustration 🡪 Child negative affect | **.023 [.013, .034]** |
| Child positive affect 🡪 Autonomy supportive behavior | **.007 [.001, .022]** |
| Child positive affect 🡪 Parental need satisfaction | **.008 [.002, .017]** |
| Child positive affect 🡪 Parental need frustration | **.008 [.002, .018]** |
| Child positive affect 🡪 Child negative affect | **.008 [.002, .016]** |
| Child negative affect 🡪 Autonomy supportive behavior | **.029 [.012, .050]** |
| Child negative affect 🡪 Parental need satisfaction | **.006 [.001, .014]** |
| Child negative affect 🡪 Parental need frustration | **.010 [.003, .022]** |
| Child negative affect 🡪 Child positive affect | **.008 [.002, .017]** |

*Note.* Table displays unstandardized regression coefficients and variances of random effects (95% credible interval in parentheses). Parameters whose 95% credible interval does not contain zero are highlighted in bold face. Note that only the results of the within-person associations are reported. For findings on the between-person level (correlations among the nine variables) see the respective Mplus output in the OSF repository (<https://osf.io/aj6fk/>)*. N* = 535.

^a^0= during the week, 1= weekend/holiday; ^b^0= no school work today, 1= school work today;

Table S8.

*Standardized Factor Loadings of the Indicators.*

| Item number | Loading |
| --- | --- |
| Cohesion |  |
| 1 | .472 |
| 11 | .482 |
| 14 | .602 |
| 17 | .505 |
| 23 | .622 |
| Expressiveness |  |
| 4^a^ | .626 |
| 5^a^ | .771 |
| 20^a^ | .582 |
| Organization |  |
| 6^a^ | .777 |
| 10^a^ | .772 |
| 19^a^ | .693 |
| 29^a^ | .417 |
| Control |  |
| 7 | .655 |
| 9 | .726 |
| 12 | .609 |
| 15 | .523 |
| 24 | .454 |
| Emotional Problems |  |
| 3 | .389 |
| 8 | .759 |
| 13 | .630 |
| 16 | .605 |
| 24 | .662 |
| Hyperactivity |  |
| 2 | .632 |
| 10 | .576 |
| 15 | .814 |
| 21^a^ | .535 |
| 25^a^ | .805 |
| Prosocial Behavior |  |
| 1 | .649 |
| 4 | .508 |
| 9 | .645 |
| 17 | .564 |
| 20 | .657 |
| Vitality |  |
| 1 | .819 |
| 2^a^ | .732 |
| 3 | .667 |
| 4 | .867 |
| 5 | .734 |
| 6 | .824 |
| 7 | .934 |
| Stress |  |
| 1 | .552 |
| 2 | .733 |
| 3 | .779 |
| 4^a^ | .565 |
| 5^a^ | .574 |
| 6 | .646 |
| 7^a^ | .557 |
| 8^a^ | .747 |
| 9 | .635 |
| 10 | .695 |

*Note.* Table depicts standardized factor loadings of all 49 items. All loadings were statistically significant, *p* < .05. *N* = 962.

Item numbers correspond to the position of the respective items in the publication from which they were drawn. Please see the codebook (<https://osf.io/aj6fk/>) for further details.

^a^ Item was recoded prior to the analyses

Table S9.

*Tests of Measurement Invariance.*

| Scale | χ² | c | df | CFI | RMSEA | SRMR | BIC |
| --- | --- | --- | --- | --- | --- | --- | --- |
|  | Family environment | | | | | | |
| Configural MI | 1016.935 | 1.068 | 480 | .911 | .049 | .079 | 39733.200 |
| Weak MI | 1044.966 | 1.069 | 493 | .909 | .049 | .080 | 39683.902 |
| Strong MI | 1061.418 | 1.067 | 506 | .908 | .048 | .081 | 39619.400 |
| Strict MI | 1066.519 | 1.080 | 523 | .910 | .047 | .083 | 39534.076 |
|  | SDQ | | | | | | |
| Configural MI | 793.768 | 1.078 | 373 | .921 | .049 | .066 | 21115.043 |
| Weak MI | 809.782 | 1.076 | 385 | .920 | .049 | .067 | 21057.200 |
| Strong MI | 824.694 | 1.073 | 397 | .920 | .048 | .067 | 20997.075 |
| Strict MI | 840.151 | 1.072 | 412 | .920 | .047 | .067 | 20920.673 |
|  | Vitality | | | | | | |
| Configural MI | 149.863 | 1.186 | 67 | .980 | .051 | .037 | 19909.237 |
| Weak MI | 161.044 | 1.159 | 73 | .979 | .051 | .040 | 19881.183 |
| Strong MI | 172.230 | 1.145 | 79 | .978 | .050 | .041 | 19854.873 |
| Strict MI | 174.906 | 1.157 | 86 | .979 | .047 | .042 | 19816.968 |
|  | Stress | | | | | | |
| Configural MI | 400.183 | 1.154 | 157 | .929 | .058 | .047 | 34109.961 |
| Weak MI | 413.112 | 1.144 | 166 | .928 | .056 | .049 | 34065.532 |
| Strong MI | 438.695 | 1.137 | 175 | .923 | .057 | .050 | 34036.361 |
| Strict MI | 449.910 | 1.135 | 185 | .923 | .055 | .054 | 33986.779 |

*Note.* *N* = 468. c = scaling factor; df = degrees of freedom; CFI = comparative fit index; RMSEA = root mean square error of approximation; SRMR = standardized root mean square residual; BIC = Bayesian information criterion; MI = measurement invariance.

Table S10.

*Latent Difference Score Model: Intercorrelations of Latent Variables.*

|  |  | Correlations | | | | | | | | | | | | | | | | |
| --- | --- | --- | --- | --- | --- | --- | --- | --- | --- | --- | --- | --- | --- | --- | --- | --- | --- | --- |
|  |  | 2 | 3 | 4 | 5 | 6 | 7 | 8 | 9 | 10 | 11 | 12 | 13 | 14 | 15 | 16 | 17 | 18 |
| 1 | Cohesion | -.06 | .49* | .04 | .34* | .03 | -.05 | -.25* | -.13* | -.10 | -.29* | -.10 | .44* | -.08 | .30* | -.01 | -.35* | -.01 |
| 2 | Change Cohesion |  | .02 | .52* | -.22* | .40* | -.12 | .52* | -.14 | -.05 | -.08 | -.14 | -.04 | .34* | -.05 | .35* | .03 | -.38* |
| 3 | Expressiveness |  |  | -.18 | .27* | -.06 | -.00 | .00 | -.18* | -.01 | -.11* | -.00 | .14* | .10 | .20* | -.05 | -.15* | -.02 |
| 4 | Change Expressiveness |  |  |  | -.12 | .37* | -.04 | -.01 | .10 | .05 | .07 | -.31* | -.01 | .21 | -.14* | .17* | .00 | -.14 |
| 5 | Organization |  |  |  |  | -.09 | .48* | .06 | -.22* | .09 | -.26* | .16* | .30* | -.15 | .22* | -.03 | -.25* | .06 |
| 6 | Change Organization |  |  |  |  |  | -.10 | .26 | .10 | -.19* | -.00 | -.20 | -.09 | .14 | -.05 | .06 | .05 | -.20* |
| 7 | Control |  |  |  |  |  |  | -.05 | -.07 | .07 | -.02 | .01 | .11 | -.03 | .14* | -.01 | -.07 | .17* |
| 8 | Change Control |  |  |  |  |  |  |  | -.16 | .08 | -.04 | .16 | -.02 | .05 | -.02 | -.01 | -.01 | -.02 |
| 9 | Emotional Problems |  |  |  |  |  |  |  |  | -.34* | .44* | -.18* | -.23* | -.07 | -.36* | -.02 | .43* | -.10 |
| 10 | Change Emotional Problems |  |  |  |  |  |  |  |  |  | -.10 | .43* | .04 | -.20 | -.04 | -.13 | -.02 | .29* |
| 11 | Hyperactivity |  |  |  |  |  |  |  |  |  |  | -.24* | -.50* | .09 | -.44* | .02 | .41* | -.07 |
| 12 | Change Hyperactivity |  |  |  |  |  |  |  |  |  |  |  | .14 | -.61* | .09 | -.14 | -.03 | .21* |
| 13 | Prosocial behavior |  |  |  |  |  |  |  |  |  |  |  |  | -.29* | .33* | -.07 | -.37* | .13* |
| 14 | Change Prosocial behavior |  |  |  |  |  |  |  |  |  |  |  |  |  | .01 | .20* | -.02 | -.22* |
| 15 | Vitality |  |  |  |  |  |  |  |  |  |  |  |  |  |  | -.35* | -.67* | .18* |
| 16 | Change Vitality |  |  |  |  |  |  |  |  |  |  |  |  |  |  |  | .14* | -.41* |
| 17 | Stress |  |  |  |  |  |  |  |  |  |  |  |  |  |  |  |  | -.41* |
| 18 | Change Stress |  |  |  |  |  |  |  |  |  |  |  |  |  |  |  |  |  |

*Note.* Table displays correlations among the latent variables in the latent difference score model. *N* = 468. **p* < .05.

**Appendix S1. Factor Structure of the Measurement Scales.**

We split the total sample of the baseline assessment (N = 970) randomly into two smaller samples of 485 participants each to examine the factor structure of the measurement instruments in two independent samples. Because the 19 items that were administered to assess family environment have previously been used to assess three dimensions of family climate (positive emotional climate, organization, control) in adolescents only, it is unknown if these items capture the same three dimensions when rated from the parents’ perspectives. Therefore, we conducted an exploratory factor analysis for the data in the first half of the total sample in a first step. To determine the number of factors to be extracted, we considered three criteria: the minimum average partial (MAP), parallel analysis, and the Bayesian information criterion (BIC). We followed up with a confirmatory factor analysis (CFA) in the same sample. Modification indices were inspected to improve model fit, if necessary. To determine the necessity of model improvement, we focused on absolute model fit indices (CFI, RMSEA, SRMR). We then validated the chosen model using a CFA in the second sample. The other three scales (SDQ, PSS, vitality) have been used previously in adult samples. We therefore conducted separate CFAs for the three instruments in the first sample and modified the measurement models based on the modifications indices, if indicated. The models were then validated in the second (independent) sample.

In the first step, the 19 items assessing family environment were analyzed in an exploratory factor analysis in half of the baseline sample. One item was uncorrelated with four out of eight other items of its designated factor (positive emotional climate); a second item from the organization subscale exhibited higher loadings on the control subscale in all models. These two items were therefore removed and the remaining 17 items were used for the following analyses only (oblimin rotation). There was no clear indication as to how many factors should be retained; depending on the criterion, optimal number of factors was two (MAP) or four (BIC, parallel analysis). In the two factor solution, the two factors organization and control were collapsed, while the other items loaded on one common factor (positive emotional climate). In the four factor solution, organization and control formed separate factors and positive emotional climate was split into two factors. Based on the item contents, we termed these two factors cohesion (items 1, 11, 14, 17, and 23) and expressiveness (items 4, 5, and 20), in line with the terminology used by Moos (1990). Factor loadings and factor correlations of the four factor solution are reported in Table S5. A confirmatory factor analysis conducted in this sample showed adequate (RMSEA = .076) to subpar fit (CFI = .831, SRMR = .080) of this four factor model. Modification indices suggested adding a residual covariance between two indicators of the cohesion factor. Adding this covariance improved model fit, RMSEA = .062, SRMR = .067, CFI = .888. Fit indices of this four factor model in the second (independent) sample suggested adequate fit, RMSEA = .056, SRMR = .065, CFI = .900. Because the four factor model was preferred by two out of three indices in the EFA, yielded adequate fit in the CFA, and was better interpretable from a theoretical perspective than the two factor model, a four factor model for the 17 remaining items assessing family environment was assumed for all analyses.

For the remaining three instruments (SDQ, vitality, PSS), we conducted individual CFAs in the first subsample (the same participants for whom we conducted the exploratory factor analysis for family environment). Results (see Table S6) indicated moderate to good model fit in these analyses. To further improve model fit, we investigated modification indices, which indicated that for each of the three models, estimating one error covariance might further improve model fit. We validated these measurement models in the second subsample, which resulted in acceptable to good model fit for all scales. We note that fit for the PSS was below our acceptable levels for two out of three indicators in Sample 2 (RMSEA =.107, CFI = .871, SRMR = .055), but acceptable in Sample 1 (RMSEA =.088, CFI = .916, SRMR = .048).

**Appendix S2. Measurement Invariance.**

We tested measurement invariance across time (from baseline to post assessment) in the sample who completed both assessments. To that end, we compared a series of nested models: (1) configural measurement invariance (same factor structure at both measurement occasions, all model parameters were unique at both measurement occasions); (2) weak measurement invariance (configural measurement invariance + factor loadings constrained to equality across measurement occasions); (3) strong measurement invariance (weak measurement invariance + item intercepts constrained to equality across measurement occasions); (4) strict measurement invariance (strong measurement invariance + item residual variances constrained to equality across measurement occasions). Measurement invariance was tested by sequentially comparing the more restrictive model to the less restrictive model. Models were compared based on absolute fit indices (CFI, RMSEA, SRMR), the BIC, as well as an adjusted likelihood ratio test comparing the nested models (Yuan & Bentler, 2008). Model comparisons were implemented via the MplusAutomation package (Hallquist, & Wiley, 2018) in R. Indicator specific covariances across time were estimated in all models.

In these analyses. the latent variables were scaled via the effects-coding approach suggested by Little, Slegers, and Card (2006). That is, means and variances of all latent variables were freely estimated, factor loadings were estimated with the constraint that they sum up to the number of indicators of the factor, and intercepts of the indicators were estimated under the constraint that they sum up to zero. This scaling procedure has been suggested to circumvent the necessity to choose a reference indicator for each factor which can lead to erroneous conclusions when testing for measurement invariance.

Results for all these models are reported in Table S11. All nested model comparisons for SDQ and vitality were not statistically significant, *p* > .093 for all. For family environment (stress), the weak (strong) invariance model fitted the data statistically significantly worse than the corresponding configural (weak) invariance model, χ²(13) = 28.02, *p* = .009 (χ²(9) = 25.99, *p* = .002). However, drop in absolute fit was negligible, and the BIC favored the more parsimonious model in both comparisons. Therefore, we considered the models assuming strict measurement variance as tenable for all constructs, and we assumed strict measurement invariance in all following models.
